# Nitric Oxide (NO) as a Reagent for Topochemical Framework Transformation and Controlled NO Release in Covalent Organic Frameworks

**DOI:** 10.1021/jacs.2c11967

**Published:** 2023-03-28

**Authors:** Sebastian T. Emmerling, Johannes Maschita, Bettina V. Lotsch

**Affiliations:** † Nanochemistry Department, Max Planck Institute for Solid State Research, Heisenbergstraße 1, 70569 Stuttgart, Germany; ‡ Department of Chemistry, University of Munich (LMU), Butenandtstraße 5-13, 81377 Munich, Germany; § E-conversion and Center for Nanoscience, Lichtenbergstraße 4a, 85748 Garching, Germany

## Abstract

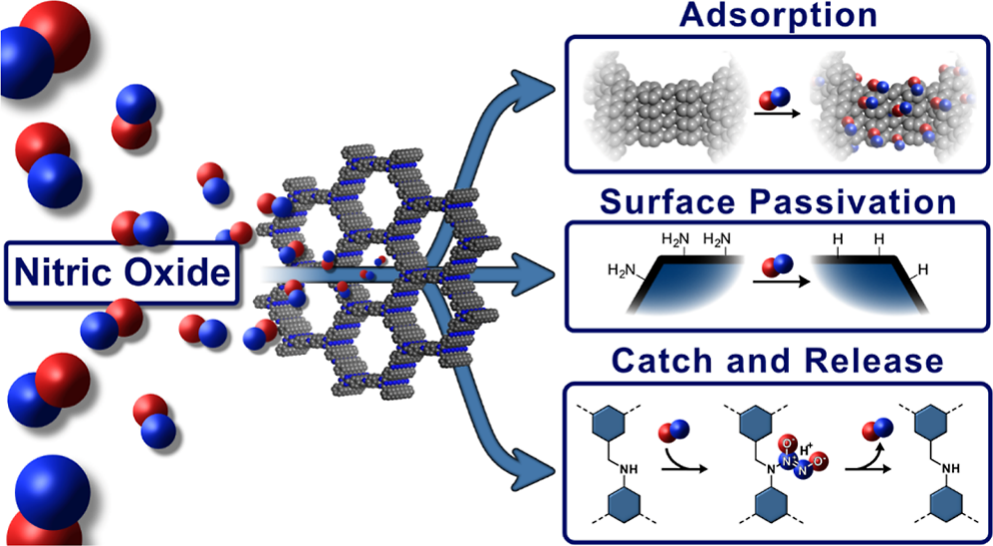

Covalent organic frameworks (COFs) have emerged as versatile platforms for the separation and storage of hazardous gases. Simultaneously, the synthetic toolbox to tackle the “COF trilemma” has been diversified to include topochemical linkage transformations and post-synthetic stabilization strategies. Herein, we converge these themes and reveal the unique potential of nitric oxide (NO) as a new reagent for the scalable gas-phase transformation of COFs. Using physisorption and solid-state nuclear magnetic resonance spectroscopy on ^15^N-enriched COFs, we study the gas uptake capacity and selectivity of NO adsorption and unravel the interactions of NO with COFs. Our study reveals the clean deamination of terminal amine groups on the particle surfaces by NO, exemplifying a unique surface passivation strategy for COFs. We further describe the formation of a NONOate linkage by the reaction of NO with an amine-linked COF, which shows controlled release of NO under physiological conditions. NONOate-COFs thus show promise as tunable NO delivery platforms for bioregulatory NO release in biomedical applications.

## Introduction

Beginning in the late 1980s, nitric oxide (NO) gained increasing interest in biological research after its key role as a signal molecule in various physiological processes in the human body was discovered.^1^ While NO plays a significant role in human health by regulating blood pressure, wound healing, and neurotransmission,^2−5^ it is more widely known as a (problematic) component of NO_*x*_ found in exhaust gases of combustion engines.^6^ With increasing road traffic, agriculture productivity, or electricity generation, artificial NO pollution became almost omnipresent in the environment around us.^7−9^ However, in contrast to the essential NO needed for physiological processes, in which NO rarely exceeds an internal concentration of 5 nM, the artificial presence of NO in our external environment can have adverse effects on human health.^10−12^ The combustion-based NO_*x*_ emissions count as major air pollutants and as a gaseous precursor of fine particulate matter (PM_2.5_), which is considered a leading environmental health risk factor, associated with 3 to 4 million premature deaths each year and a significantly reduced life expectancy.^13^ Environmental and healthcare organizations like the World Health Organization (WHO) repeatedly appeal for a reduction of air pollution and readjust their guidelines for PM_2.5_ to lower levels.^14^ Current strategies to reduce NO emissions are based on rare earth metal three-way catalysts, as found in automobiles, which reduce NO to nitrogen while simultaneously oxidizing noxious CO and hydrocarbons to CO_2_.^15^ Other strategies focus on capture and release systems, “washing” the exhaust gas by binding NO onto functional groups by forming nitrosamines, *N*-diazeniumdiolates (NONOates), and nitroso-metal complexes.^16,17^ The reversible formation of these species allows the subsequent controlled release and recycling of NO and can also be the basis of various pharmaceuticals.^18,19^

Recent advances in heterogeneous systems for NO release were made including surface-crafted polymers and silica particles with exposed NO-binding functional groups.^20−22^ Lately, highly porous materials were discovered as candidates for NO removal via adsorption and chemisorption or as catalyst/catalyst support materials for NO decomposition.^23^ Metal organic frameworks (MOFs) have been developed as NO release materials utilizing either unsaturated and open metal sites or implemented amino functionalities to adsorb NO in their pores.^1,24,25^

Covalent organic frameworks (COFs) are recent additions to the class of highly porous framework materials.^26^ Due to their high specific surface area, defined structure, high modularity, and low density, COFs could be promising candidates for NO adsorption/separation applications or as heterogeneous catalyst/catalyst support materials for NO decomposition.^27−29^

Conductive COFs have already been successfully used in chemiresistive sensor devices to detect NO and other harmful gases in the ppb range.^30,31^

Despite the possible applicability of COFs as functional materials for such applications, there remain concerns regarding their stability. According to the “COF-trilemma”, frameworks assembled by reversible covalent bond formation often suffer from low stability; therefore, their stability against the reactive NO gas might be limited at these crucial points of connection. Especially early introduced and well-established linkages like boronic esters or imine bonds suffer from instability against harsher chemical conditions.^32^ However, to tackle this trilemma, in recent years, numerous novel linkages, post-synthetic-modifications, and other stabilization strategies for COFs have been developed, broadening our toolbox to design materials that can be suitable for NO adsorption.^33−37^

In this work, we determine the stability of four different COF linkages—imine, amine, thiazole, and imide—against NO exposure and examine their suitability for NO separation applications by calculating their specific selectivity against N_2_ and CO_2_. By targeted ^15^N enrichment at the crucial COF linkages, we investigate and identify chemical modifications in the COFs’ frameworks induced by NO. These chemical modifications, including a novel type of linkage, are evaluated for their potential applications in COF chemistry and biomedical research.

## Results and Discussion

**Figure 1:**
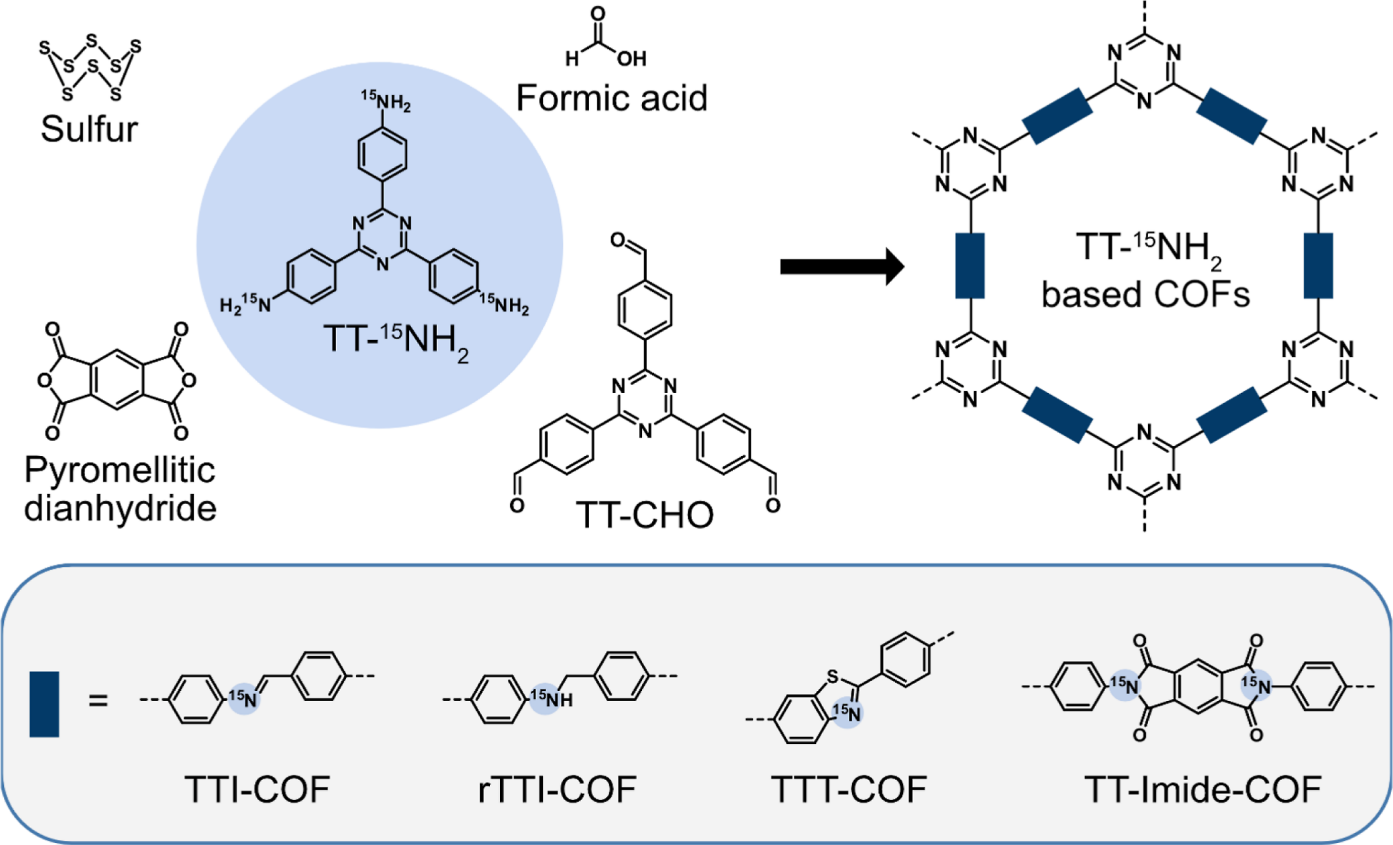
Synthesis of the four ^15^N-enriched COFs, the TTI-COF, rTTI-COF, TTT-COF, and TT-imide-COF, bearing imine, amine, thiazole, and imide linkages, respectively. All COFs are based on an enriched 4,4′,4″-(1,3,5-triazine-2,4,6-triyl)trianiline-^15^N linker, TT-^15^NH_2_, which was synthesized starting from 4-cyanobenzoyl chloride and ^15^NH_4_Cl (Scheme S1) in a three-step procedure with a good overall yield of 12.6%.

To study the effect of NO on COFs, we synthesized four ^15^N-enriched frameworks, namely, TTI-COF, rTTI-COF, TTT-COF, and TT-Imide-COF (Figure 1),^33,37−39^ which are all based on an enriched 4,4′,4″-(1,3,5-triazine-2,4,6-triyl)trianiline-^15^N linker (TT-^15^NH_2,_Figure 1). The isotope enrichment of the COFs enhances the sensitivity in ^15^N cross-polarization magic angle spinning solid-state nuclear magnetic resonance (CP-MAS ssNMR) spectroscopy and thus allows a detailed analysis of the respective linkages. This is essential since we expect these nitrogen sites to be the most reactive groups toward NO and therefore to constitute the most interesting moieties in the COFs during this study.

### NO Uptake

**Figure 2:**
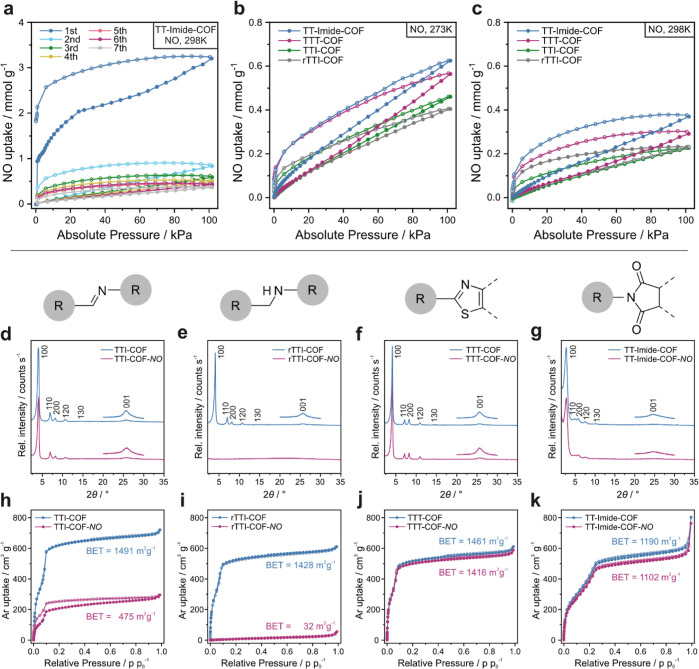
(a) NO adsorption cycles of TT-Imide-COF. Comparison of stabilized NO adsorption of TTI-COF, rTTI-COF, TTT-COF, and TT-Imide-COF at (b) 273 K and (c) 298 K after completing the 7th run. PXRD and argon sorption measurement at 87 K of TTI-COF (d,h), rTTI-COF (e,i), TTT-COF (f,j), and TT-imide-COF (g,k) before (blue) and after (red) NO exposure. Filled circles represent the adsorption, and empty circles represent the desorption.

NO sorption measurements on ^15^N-enriched COFs were performed starting at 298 K. In all cases, an initial high but only partially reversible adsorption capacity was found within the first adsorption cycle. In this first measurement, COF-dependent uptakes between 1.5 and 6 mmol g^–1^ NO were observed. In addition, a steep adsorption slope below 2 kPa for TT-Imide-COF and TTI-COF (Figures 2a and S29), around 30 kPa for TTT-COF (Figure S33), and around 90 kPa for rTTI-COF was found (Figure S31). The steep uptake within the first cycle at different pressure points for each COF indicates differing mechanisms for the irreversible chemisorption of NO. Thus, in each case, the hysteresis does not close to the initial point during the first measurement. Multiple adsorption and desorption cycles at 298 K show a strong decrease in NO uptake after the first cycle toward a fraction of the initial uptake. This trend of an initial high and irreversible NO uptake and its strong decline in further cycles indicates a chemisorption process of NO on the frameworks, consistent with a progressive quenching of the reactive sites.

For all frameworks, the decline in the irreversible uptake of NO toward the second cycle is the most prominent, while the following sorption cycles show only a small decrease, which stabilizes after approximately three cycles (Figures S30, S32, S34, and S35). To ensure reproducible NO physisorption data at different temperatures, all COFs were cycled seven times to ensure stabilization of the systems before measurements at 273, 288, and 298 K were performed (Figures 1b,c and S36). In contrast to the initial NO sorption cycles, subsequent measurements show fully reversible isotherms for all four COFs, indicating a saturation of the systems. Further, they reveal an unusual hysteresis due to delayed NO desorption visible for all temperatures and COFs.

The measured NO physisorption capacities at 298 K—ranging between 0.2 and 0.4 mmol g^–1^ as compared to 1.5–6 mmol g^–1^ during the initial uptake—are in good agreement with the values found for simulated NO adsorption isotherms for boronate ester-based COF-105 and COF-108 by Wang et al.^40^ Note that experimental values on NO sorption in COFs have not yet been reported.

Since the NO sorption measurements suggest a reaction of NO with the frameworks, we investigated the reactions occurring during contact with the highly reactive gas. At first, the stability of the COFs toward NO was evaluated by examining the materials after exposure. These will be labeled with −*NO* further on.

Powder X-ray diffraction (PXRD) analysis of the samples reveals that TTI-COF-*NO* becomes slightly less crystalline after NO exposure and rTTI-COF-*NO* turns amorphous (Figure 2d,e). The Brunauer–Emmett–Teller (BET) specific surface area (*S*_BET_) and specific pore volume (*V*_P_) decrease for the imine-COF (Figure 2h) from *S*_BET_ = 1491 m^2^ g^–1^ (*V*_P_ = 0.917) to less than a third for the post-NO imine-COF with *S*_BET_ = 475 m^2^ g^–1^ (*V*_P_ = 0.376). The amine-COF rTTI-COF (Figure 2i) with initially *S*_BET_ = 1428 m^2^ g^–1^ (*V*_P_ = 0.778) becomes non-porous after NO exposure with *S*_BET_ = 32 m^2^ g^–1^ (*V*_P_ = 0.071 cm^3^ g^–1^). For TTT-COF and TT-imide-COF (Figure 2g, f) no changes in crystallinity after exposure are noticeable and the *S*_BET_ (Figure 2j,k) and pore volume of the samples decrease just by small amounts from 1461 m^2^ g^–1^ (*V*_P_ = 0.778 cm^3^ g^–1^) to 1416 m^2^ g^–1^ (*V*_P_ = 0.750 cm^3^ g^–1^) and from 1190 m^2^ g^–1^ (*V*_P_ = 0.802 cm^3^ g^–1^) to 1102 m^2^ g^–1^ (*V*_P_ = 0.744 cm^3^ g^–1^), respectively. For the latter, the Fourier transform infrared (FT-IR) spectra before and after NO exposure show no difference (Figures S8 and S9). However, TTI-COF-*NO* displays a new band at 1699 cm^–1^, a typical range for an aldehyde HC=O vibration, while otherwise remaining unchanged (Figure S6). The spectrum of rTTI-COF-*NO* shows significant changes compared to pristine rTTI-COF, the most prominent is the appearance of three distinct bands at 1700, 1084, and 916 cm^–1^ (Figure S7). We also observed a color change of the sample after NO exposure. UV–vis measurements reveal slightly altered absorption spectra for the post-NO COFs (Figures S20–S23). The most obvious color changes were perceived for TTI-COF and rTTI-COF– both changing from yellow to brownish color. This analysis reveals that the thiazole and imide linkages are largely inert against NO exposure since the structural integrity and porosity of the respective COFs remain unaffected. In contrast, the imine and amine linkages seem to react to a varying degree with the gas, indicated by changes in the FT-IR spectra, resulting in a loss of structural integrity and porosity.

Narrowing down suitable COF linkages for NO adsorption and separation applications to thiazole and imide functionalities, we further investigated the physisorption performance and calculated the selectivity for NO compared to that for carbon dioxide by applying the ideal adsorption solution theory (IAST). Using the post-NO CO_2_ adsorption isotherms as a reference, we calculated the initial heats of adsorption, *Q*_st_, adsorption capacities, and IAST selectivity over nitrogen for CO_2_ and NO (Table S1). For the selectivity calculations, we chose a 15/85 gas mixture for CO_2_/N_2_, as commonly found in the literature,^41^ and a 3/97 gas mixture for NO/N_2_, due to the low abundance of NO in exhaust gas mixtures.^42^ We calculated the IAST selectivities of CO_2_ over nitrogen for TT-Imide-COF-*NO* and TTT-COF-*NO* as 9.22 and 6.44, respectively. The IAST selectivity of NO over nitrogen were calculated to be 5.55 for the TT-Imide-COF-*NO* and 3.95 for TTT-COF-*NO*. Furthermore, the pressure-dependent selectivity of a binary CO_2_/NO (50/50) gas mixture was calculated (Figure S37c). Overall, our results show that imide- and thiazole-linked COFs exhibit remarkable resistance to NO gas and are promising platforms for NO gas capture or separation. Moreover, these systems show significant IAST selectivity of NO uptake over N_2_.

### Solid-State NMR Analysis

Next, we turn our attention to the imine and amine linkages that are more susceptible to NO exposure. To deconvolute the irreversible chemisorption phenomenon during the first NO adsorption cycles and to gain insights into possible reactions involved, we performed ^13^C and ^15^N CP-MAS ssNMR experiments. Utilizing the high sensitivity of our ^15^N-enriched COFs, we were able to capture even less-sensitive species like the thiazole nitrogen and minor framework defects, as is discussed in the following.

**Figure 3:**
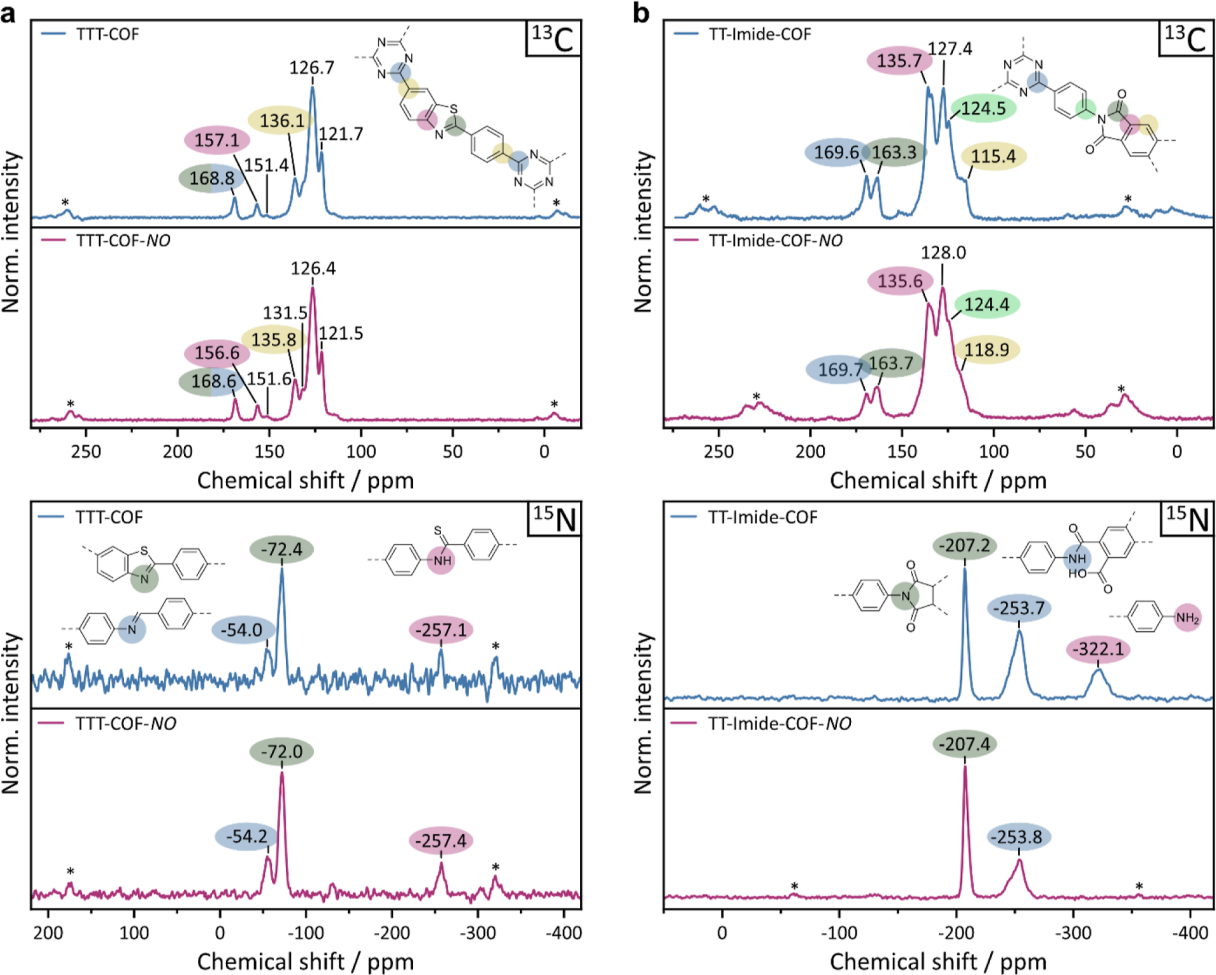
^13^C CP-MAS (top row) and ^15^N CP-MAS (bottom row) ssNMR spectra of (a) TTT-COF (blue) and TTT-COF-NO (red) and (b) TT-Imide-COF (blue) and TT-Imide-COF-NO (red).

As one of the NO resistant frameworks, TTT-COF-*NO* shows no visible changes in its ^13^C and ^15^N spectra compared to the spectrum of the pristine TTT-COF (Figure 3a). Interestingly, the high sensitivity of our ^15^N measurements reveals small amounts of imine residuals at −54 ppm (Figure 3a, bottom) that were not transformed into thiazole during the topochemical conversion. Another unexpected peak was found at −257 ppm, which we assign to a thioamide, the proposed intermediate of the topochemical conversion.^33^ It should be noted that the intensities of both moieties are more pronounced in the CP-MAS experiments than that of thiazole due to polarization transfer from neighboring protons. The related signal for the imine bond in the ^13^C CP-MAS ssNMR spectrum, expected at 151 ppm,^33^ is barely visible. No obvious signs of the increased initial NO uptake are visible. Considering the relatively minor irreversible NO uptake of 1.5 mmol g^–1^ compared to that of the other COFs (3–6 mmol g^–1^), reactions of NO with remaining oligomer impurities, sulfur and/or remaining imine bonds, as observed for TTI-COF (infra vide), are plausible.

**Figure 4:**
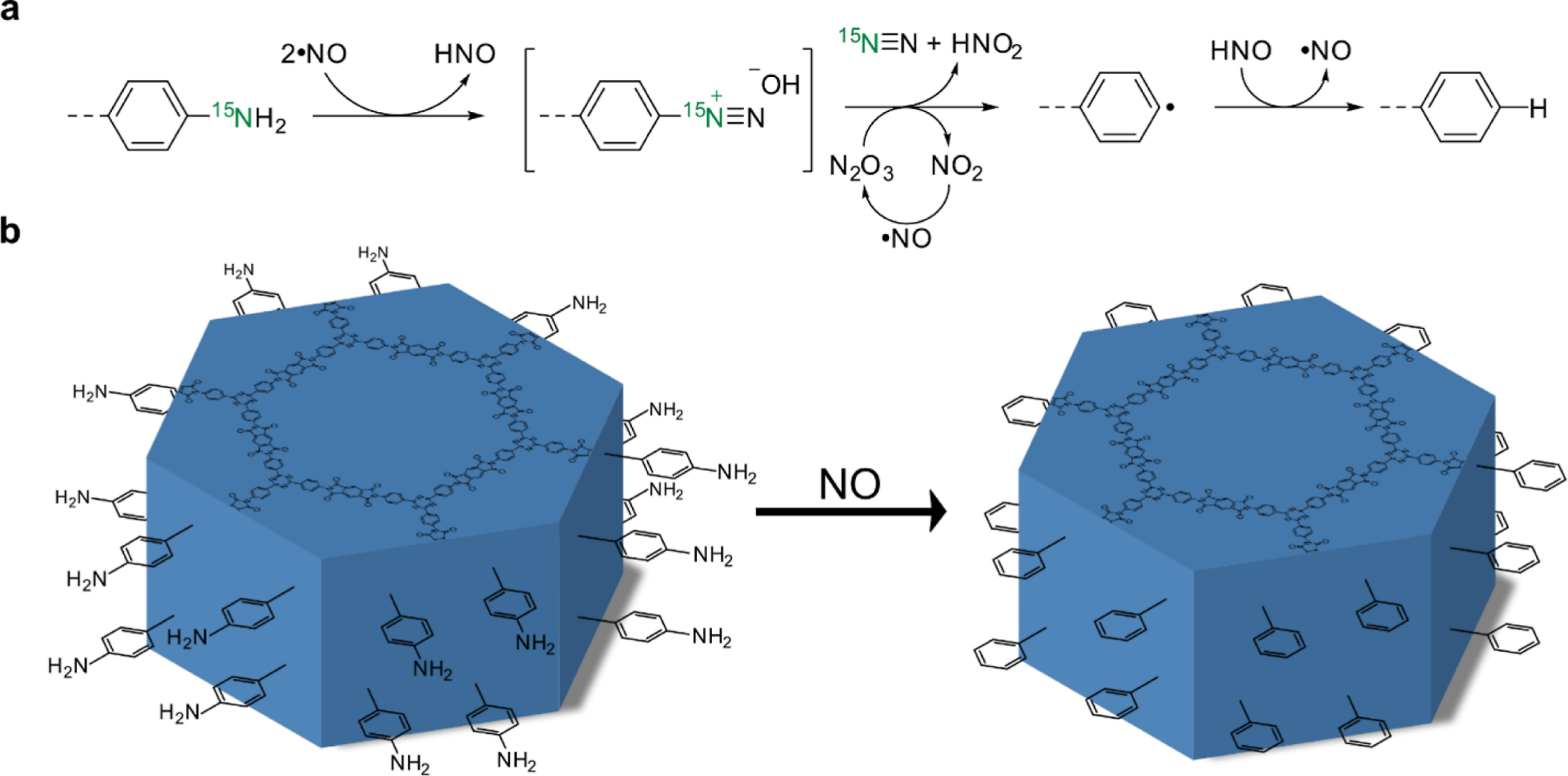
(a) Proposed mechanism for deamination of arylamines upon NO exposure. First, a diazonium salt is formed in the reaction of the free amine with NO. This salt further reacts with catalytic amounts of N_2_O_3_ impurities in the NO gas, cleaving the diazonium moiety as dinitrogen and nitrous acid. The resulting phenyl radical is subsequently quenched by exposure to the nitroxyl released in the first step or any other proton-bearing volatile compound, like H_2_O.^43^ (b) Schematic representation of crystal surface passivation of terminal amines by NO-induced deamination.

The ^13^C spectrum of TT-Imide-COF-*NO* resembles the spectrum of the pristine COF (Figure 3b, top). A minor peak broadening is observed, which might be a result of the slightly increased structural disorder of the framework. The ^15^N-CP-MAS spectrum of TT-imide-COF reveals three signals at −207, −254, and −322 ppm, assigned to the imide, the intermediate amic acid, and the remaining terminal amine nitrogen at the crystallite edges, respectively (Figure 3b, bottom). However, only the imide signal is observed in a (quantitative) direct excitation experiment, suggesting that the proton-bearing species constitute a minor side phase and are overemphasized due to their sensitivity in CP-MAS experiments (Figure S11). Upon exposure to NO, the signal at −322 ppm, assigned to terminal amine groups, vanished completely, giving the first direct indication for a chemical reaction of the framework with the adsorbate. Furthermore, the relative intensity of the amide at −254 ppm is lowered compared to that of the imide at −207 ppm after NO treatment. Lacking any new signals, we suspect a clean conversion of the amines by NO, passivating the crystallite surface edges, according to a proposed denitrogenation radical reaction mechanism depicted in Figure 4 based on findings of Itoh et al. on the deamination of aniline.^43^

First, a diazonium salt is formed in the reaction of the free amine with NO. This salt further reacts with catalytic amounts of N_2_O_3_ impurities in the NO gas, cleaving the diazonium moiety as dinitrogen and nitrous acid. The resulting phenyl radical is subsequently quenched by exposure to the nitroxyl released in the first step or any other proton-bearing volatile compound, like H_2_O. Due to the deamination of the enriched ^15^N, the amine peak disappears and no new signal is visible. We envision that this interaction between the free amine groups and the NO gas could be used as a rational surface passivation strategy to purposefully remove the remaining and potentially reactive amine sites. Clearing the COF’s surface from terminal amines could be of particular interest when their chemical reactivity or electronic properties hinder or alter the targeted properties of the framework (e.g., in catalysis), especially when exposed at the crystal surface.

A reaction with the remaining amic acid intermediates remains uncertain. While a slight decline in the relative intensity compared to that of the imide signal is observed, only a single case of a reaction of NO with primary amides (under strongly basic conditions) has been reported in the literature, making at least a quantitative reaction unlikely in this case.^44^

**Figure 5:**
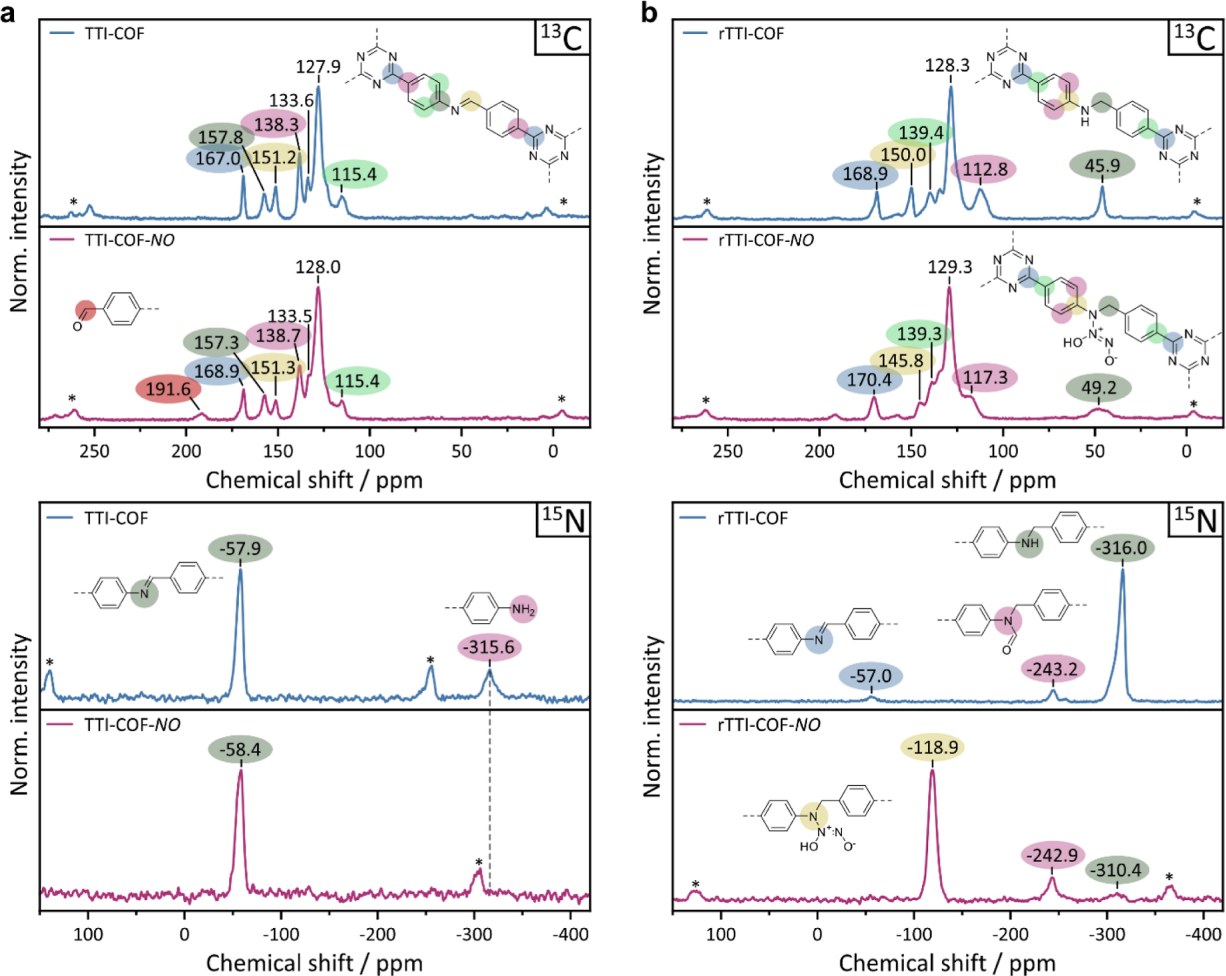
^13^C CP-MAS (top row) and ^15^N CP-MAS (bottom row) ssNMR spectra of (a) TTI-COF (blue) and TTI-COF-NO (red) and (b) rTTI-COF (blue) and rTTI-COF-NO (red).

Judging from the diffraction and sorption results, we expect more significant changes in the ssNMR spectra of TTI-COF and rTTI-COF. Indeed, TTI-COF shows a slight broadening of the peaks in the ^13^C CP-MAS NMR spectrum after NO exposure and a new signal at 191 ppm appears (Figure 5a, top). This peak corresponds to the proposed aldehyde, visible in the FT-IR spectra as a new band at 1699 cm^–1^. In parallel, a reduction of the imine signal intensity at 151 ppm compared to the remaining signals is observed, which is especially visible when comparing to the neighboring signal at 157 ppm. These findings indicate a partial cleavage of imine bonds, resulting in exposed aldehyde groups. The ^15^N CP-MAS NMR spectrum of TTI-COF-*NO* exhibits the same removal of terminal amine groups (Figure 5a, bottom), visible at −316 ppm, by deamination after contact with NO, as found for TT-Imide-COF (Figure 4). The imine signal at −58 ppm remains with minor peak broadening, as do the ^13^C signals. We rationalize these observations with an incipient decomposition according to a reaction mechanism upon NO contact shown in Scheme S2: by a [2 + 2] cycloaddition of NO to the imine bond, as described by Hrabie et al. for Schiff bases,^45^ the adduct is cleaved into the free aldehyde, as observed by the ^13^C signal at 191 ppm, and a diazo radical. Subsequently, an aryl radical is formed by the elimination of nitrogen, which is ultimately quenched.

**Figure 6:**
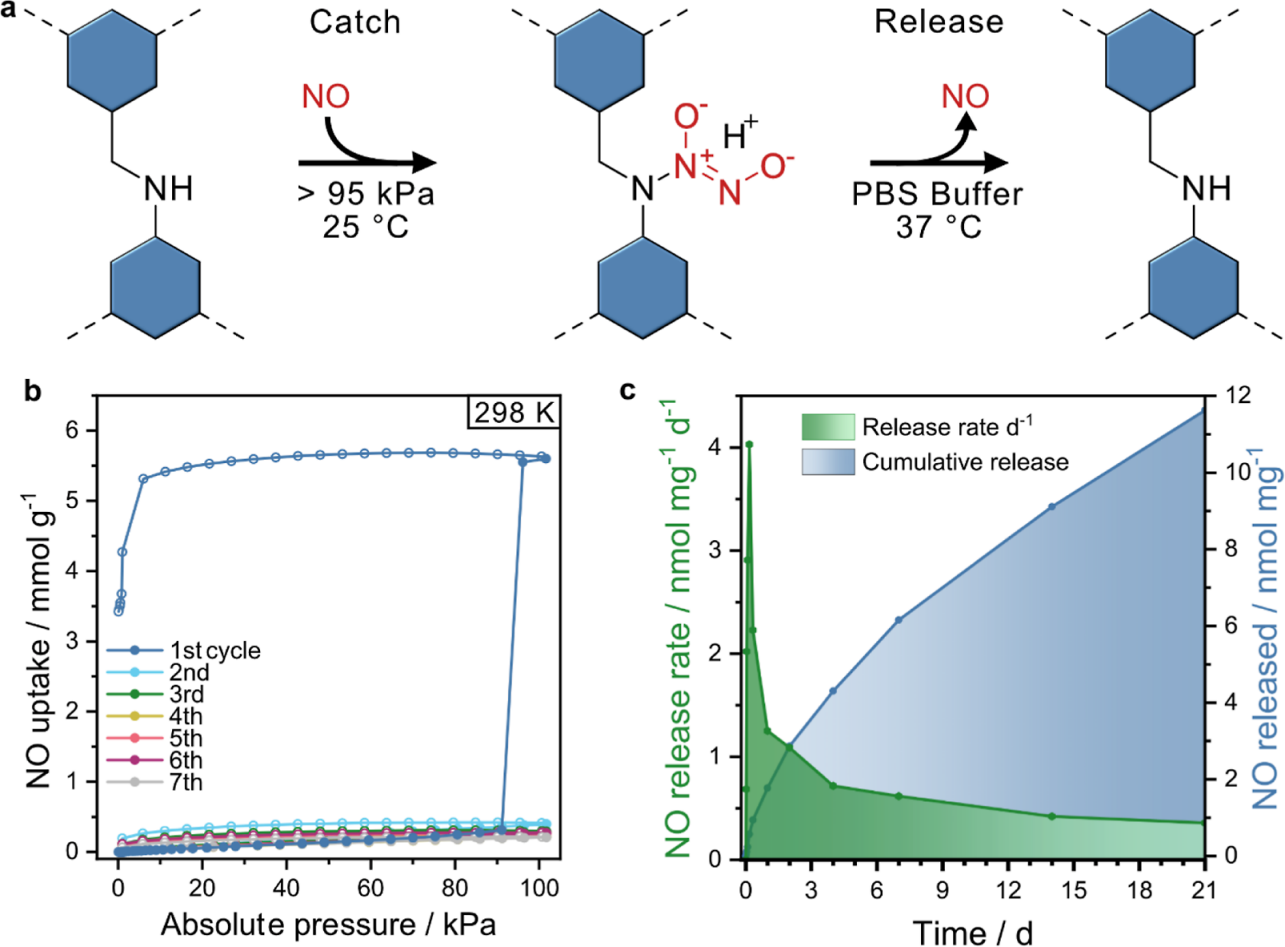
(a) Catch and release steps of the secondary amine–COF linkage. (b) NO adsorption cycles of rTTI-COF. (c) NO release and estimated release rate of rTTI-COF-NO in a pH 7.4 PBS buffer solution monitored by the Griess assay.

rTTI-COF-*NO* shows a strong broadening of all signals in the ^13^C spectrum (Figure 5a, top). Especially, the benzylic quaternary carbon at 49 ppm broadens and almost disappears into the background. This observation reflects the disorder introduced into the framework and is in good agreement with the amorphization and loss of long-distance order seen in the PXRD pattern (Figure 2e). The ^15^N spectrum reveals a significant shift of the ^15^N signal of the linkage (Figure 5b, bottom). The secondary amine-related peak of rTTI-COF at −316 ppm shifts to −119 ppm for rTTI-COF-*NO*, indicating a complete transformation of the bond. This novel linkage is identified as *N*-diazeniumdiolate (NONOate), a species that is also observed during NO adsorption in amine-functionalized MOFs and is formed by the addition of 2 equiv of NO to the amine linkage of rTTI-COF at 95 kPa NO pressure (Figure 6a,b).^1,24^ The post-synthetic linkage modification is mirrored in the FT-IR spectrum by the appearance of three new, distinct bands at 1700, 1084, and 916 cm^–1^ as mentioned above (Figure S7). The complete disappearance of the ^15^N-amine indicates full conversion of the amine into the NONOate linkage. Introducing such polar, bulky NONOate groups adds both steric and electrostatic repulsion between the layers, thus forcing the layers to distort and shift, resulting in the observed loss of long-range order, layer integrity, and porosity. Besides this drawback, the described simple gas phase modification of amine-linked COFs by NO introducing NONOate groups displays exciting new possibilities for the design of polymers and COFs bearing novel linkages and functionalities.

### NO Release

Along these lines, implementing NONOate functional groups hold great promise toward COFs as NO releasing delivery platforms for bioregulatory NO release in therapeutic or medicinal chemistry applications (Figure 6a).^5,46,47^ NO release under physiological conditions was tested by suspending rTTI-COF-*NO* in a pH 7.4 PBS buffer solution at 37 °C and monitoring the NO release using a Griess assay.^48^ The NO release profile of rTTI-COF-*NO* shows an exponential release behavior with an overall very slow but steady release of the chemisorbed NO over several days (Figure 6c). An initial faster release over the first 24 h of around 1.8 nmol NO is observed, followed by a long period of 3 weeks in which another 9.9 nmol NO is released. After NO release, crystallinity and specific surface area are partially recovered (Figures S5 and S40), evidencing the successful rearrangement of the regenerated amine-linked domains into an ordered structure. While the total amount per gram of NO delivered by the COF is significantly lower than that of the NO chemisorbed in recently published MOFs or polymers,^23,46,49^ they are well in the concentration range of physiological processes.^10,50^ In addition, the controlled release of NO over such long periods (i.e. multiple days to weeks) sets rTTI-COF-*NO* apart from the typical rapid release over seconds to hours observed for other NONOate derivatives and suggests that NONOate COFs could be promising candidates for wound healing or related applications where a slow NO release is key.^18,50^ Unlike MOFs, COFs also do not contain heavy metals that can prevent their use in pharmaceuticals. Further, we presume that the origin of this moderate but gradual release is due to the limited diffusion of water into and NO out of the disordered and non-porous but flexible framework. We also observed a minor recovery of crystallinity, which we attribute to the recovery of the original amine linkage. Due to the small amounts of NO released during the release experiment, most of the framework remained as the NONOate-linked COF. The diffusion of water into the framework is crucial to protonate the NONOate functional groups and trigger the NO release. Hence, control over the crystallinity during linkage transformation may be used to control the rate and amount of NO released by NONOate-COFs. Crystallinity and therefore enhanced accessibility of the NONOate functional groups in the COF pores could be achieved by implementing readily available strategies to direct and lock the layer stacking, or by using 3D COFs.^51−54^

Taken together, our NMR analysis explains the different linkage stabilities and transformations and rationalizes the observed initial irreversible adsorption behavior, which is largely due to the chemical reaction of NO with terminal amine groups or linkages of the frameworks.

## Conclusions

We have identified NO as a synthetically flexible reagent for the rational topochemical framework modification in COFs. We have developed a straightforward synthesis of a ^15^N-enriched version of the commonly used TT-amine linker, which was successfully used to prepare multiple COFs with varying linkage chemistries—imine, amine, thiazole, and imide. The ^15^N-enriched linkage archetypes were then used as chemical probes to verify the existence of previously postulated terminal groups and reaction intermediates during linkage formation and modification. We demonstrated that all frameworks with unreacted terminal amine sites can be quantitatively passivated with NO by a denitrogenation reaction, leading to a clean surface defunctionalization of the frameworks. In addition, we could show that the reactivity of the different linkages toward NO is distinctly different: while imide and thiazole-linked COFs were found to be largely unreactive toward NO, imine and amine-linked COFs undergo local (imine) and global (amine) linkage conversions, leading to significant changes in the surface area and framework crystallinity. We have further demonstrated a topochemical modification of amine-linked COFs by NO, forming the novel NONOate COF linkage by a solid–gas phase reaction at room temperature. Controlled and extended NO release by this NONOate linkage was observed under physiological conditions, which opens the door to the use of amine-linked COFs as potential platforms for “NO catch and release” scenarios in biomedical applications. We have thus demonstrated that besides being toxic and highly reactive, NO is a versatile reagent for crystal surface and linkage modification, thus greatly expanding the synthetic toolbox of COF chemistry.

## Data Availability

The data that support the findings of this study are available in DaRUS with DOI: 10.18419/darus-3002.

## Supporting Information Available

Description of materials and methods, synthesis of the ^15^N-enriched TT-linker and COFs, FT-IR spectra, PXRD patterns and Pawley refinements, gas sorption isotherms, BET plots, pore size distribution plots, liquid- and solid-state NMR spectra, and transmission electron microscopy pictures (PDF)
