# Supplementary material for: Nitric Oxide (NO) as a Reagent for Topochemical Framework Transformation and Controlled NO Release in Covalent Organic Frameworks
Source: J Am Chem Soc. 2023 Mar 28;145(14):7800–9. doi: 10.1021/jacs.2c11967 (PMC10103124; doi:10.1021/jacs.2c11967)
Supplement: Supplementary file 1 — ja2c11967_si_001.pdf [file ja2c11967_si_001.pdf]

Supporting Information

NO as a Reagent for Topochemical Framework Transformation and Controlled Nitric Oxide Release in Covalent Organic Frameworks

Sebastian T. Emmerling,[a,b,⊥] Johannes Maschita,[a,b,⊥] and Bettina V. Lotsch* [a,b,c]

[a] Max Planck Institute for Solid State Research, Heisenbergstraße 1, 70569 Stuttgart, Germany

[b] Department of Chemistry, University of Munich (LMU), Butenandtstraße 5-13, 81377 München, Germany

[c] E-conversion and Center for Nanoscience, Schellingstraße 4, 80799 München, Germany

⊥ S. T. E. and J. M. contributed equally to this work.

Table of Contents

Instrumental Details and Methods - 2 -
Fourier-Transform Infrared Spectroscopy - 2 -
Transmission Electron Microscopy - 2 -
X-ray Powder Diffraction - 2 -
Nuclear Magnetic Resonance Spectroscopy - 2 -
UV/VIS Spectroscopy - 3 -
Sorption - 3 -
Explanation of fittings and selectivity calculations based on ideal adsorption solution theory (IAST) - 3 -
Materials and Synthesis - 4 -
Synthesis of 4,4',4"-(1,3,5-triazine-2,4,6-triyl)trianiline-^{15}N. - 4 -
COF Synthesis - 5 -
Synthesis of ^{15}N Enriched TTI-COF - 5 -
Synthesis of ^{15}N Enriched rTTI-COF - 6 -
Synthesis of ^{15}N Enriched TTT-COF - 6 -
Synthesis of ^{15}N Enriched TT-Imide-COF - 6 -
Exposure of COFs to NO - 6 -
NO release experiments - 7 -
Supplementary Data - 8 -
Supplementary PXRD Data - 8 -
Supplementary FT-IR Spectra - 11 -
Supplementary NMR Spectra - 14 -
Supplementary TEM Images - 18 -
Supplementary UV/VIS Spectra - 20 -
Supplementary Sorption Data - 22 -
Supplementary XPS Spectra - 33 -
Author Contributions - 34 -
References - 34 -

Instrumental Details and Methods

Fourier-Transform Infrared Spectroscopy

Fourier-transform infrared (FT-IR) spectra were recorded on a PerkinElmer UATR Two in attenuated total reflection (ATR) geometry equipped with a diamond crystal.

Transmission Electron Microscopy

Transmission electron microscopy (TEM) was performed with a Philips CM30 ST (300kV, LaB_6 cathode). The samples were prepared dry onto a copper lacey carbon grid (Plano). Images were recorded with a TVIPS TemCam-F216 CMOS camera. The program EM-Menu 4.0 Extended was used for analysis.

X-ray Powder Diffraction

X-ray powder diffraction (PXRD) measurements were performed on a Stoe Stadi-P diffractometer in Debye-Scherrer geometry with Cu-$K_{\alpha 1}$ radiation equipped with a Ge(111) primary monochromator. The glass capillaries (1 mm in diameter) were spun during data collection for an improved particle statistics. Pawley refinements of the different COF structures were performed using TOPAS V6. Model structures created by Material Studio were used for the Pawley refinements with fixed atomic coordinates. The peak profile of the XRPD patterns was described by applying the fundamental parameter approach as implemented in TOPAS. The background was modeled by Chebychev polynomials. The microstructure of the different COFs was modeled using microstrain (Lorentzian and Gaussian components).

Nuclear Magnetic Resonance Spectroscopy

Solid state nuclear magnetic resonance spectra (ssNMR) were recorded on a Bruker Avance III 400 MHz spectrometer (magnetic field 9.4 T). For ssNMR spectroscopy, the samples were packed in ZrO_2 rotors, and spun in a Bruker WVT BL4 double resonance MAS probe. The spinning rate was 12-14 kHz in ^{13}C measurements, and 6 kHz in ^{15}N experiments. A standard cross-polarization sequence with a ramped contact pulse was used for both nuclei. The duration of contact pulse was 2 ms for ^{13}C and 4 ms for ^{15}N. A total of 4096-8192 scans were routinely accumulated in ^{13}C experiments, and 80000 scans in the experiments with ^{15}N. All the measurements were performed under conditions of high-power broadband proton decoupling (SPINAL 64) with the spectral conditions being optimized for the shortest relaxation delay by measuring ^{1}H T_1 relaxation time. Chemical shifts were referenced relative

to tetramethylsilane in ^{13}C (δ_{iso} = 0.0 ppm) and relative to nitromethane in ^{15}N (δ_{iso} = 0.0 ppm), with solid glycine as the secondary reference (δ_{iso} [^{15}N] = -347.54 ppm).

UV/VIS Spectroscopy

Diffuse reflectance UV-Vis spectra were collected on a Cary 5000 spectrometer referenced to barium sulfate as reference.

Sorption

Sorption measurements were performed on a Quantachrome Instruments Autosorb iQ MP. BET suface areas and pore size distributions were calculated from argon isotherms recorded at 87 K using the quenched solid-state density functional theory (QSDFT) for cylindrical pores in carbon model for argon at 87 K. CO_2 and NO isotherms were measured at 273, 288 and 298 K.[1]

Explanation of fittings and selectivity calculations based on ideal adsorption solution theory (IAST)

The NO and CO_2 isotherms were fitted with a dual-site Langmuir-Freundlich model (Figure S36 and S37). n is the adsorbed gas amount (mmol g^{-1}), p is the pressure in the bulk gas phase (bar), q_{sat} is the saturation amount (mmol g^{-1}), b is the Langmuir-Freundlich parameter (bar), α is the Langmuir-Freundlich exponent (dimensionless) for two adsorption sites A and B.[2]

$$n = \frac{q_{sat,A} b_A p^{\alpha_A}}{1 + b_A p^{\alpha_A}} + \frac{q_{sat,B} b_B p^{\alpha_B}}{1 + b_B p^{\alpha_B}}$$

The IAST selectivities S_{IAST} were calculated with the IAST equation. SIAST is the selectivity (dimensionless), q is the adsorbed amount (mmol g^{-1}), and p is the partial pressure (bar). [2]

$$S_{IAST} = \frac{q_1/q_2}{p_1/p_2}$$

Materials and Synthesis

Chemicals. All starting materials, unless otherwise specified, were obtained from Sigma-Aldrich Chemicals, and used without further purification. All solvents, unless otherwise specified, were obtained from Acros Organics, and used without further purification. ^{15}N-Ammonium Chloride was purchased from Sigma Aldrich. 2,4,6-Tris(4-aminophenyl)-1,3,5-triazine and 4,4',4''-(1,3,5-triazine-2,4,6-triyl)tribenzaldehyde were prepared according to a literature procedure.[3]

Synthesis of 4,4',4''-(1,3,5-triazine-2,4,6-triyl)trianiline-^{15}*N*.

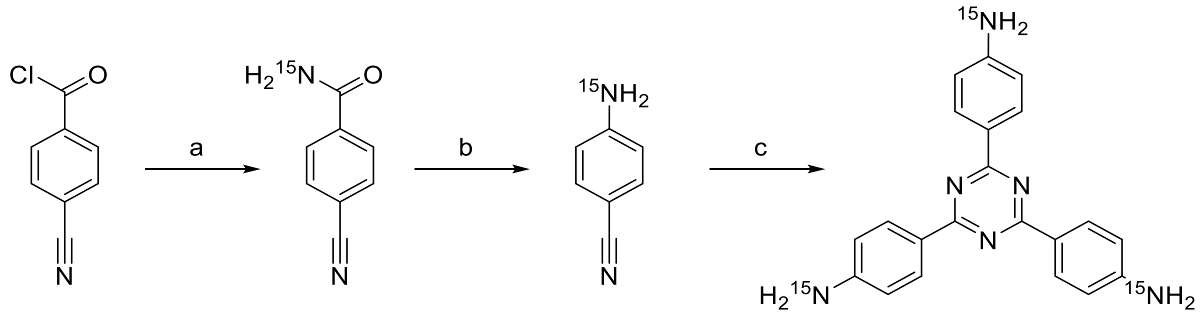

Scheme S1. Synthesis of ^{15}N-4,4',4''-(1,3,5-triazine-2,4,6-triyl)trianiline linker.

4-Cyanobenzamide-^{15}*N*. Ammonium chloride-^{15}*N* (1.81 g, 33.2 mmol) was dissolved in 12.5 mL water and the solution cooled to 0 °C. 20 mL diethyl ether was added followed by 4-cyanobenzoyl chloride (5.0 g, 20.2 mmol). After 5 minutes 5 mL aqueous sodium hydroxide (10 M) was added and the mixture stirred for 30 minutes. The white precipitate was collected by filtrations and washed with small amounts of ice cooled water. After drying the solid under air and *in vacuo*, 3.75 g (30.2 mmol, 85 %) of 4-cyanobenzamide-^{15}*N* was obtained.

^{1}H NMR (400 MHz, DMSO-*D*6) δ 8.04 – 7.99 (m, 2H), 7.97 – 7.91 (m, 2H), 7.56 (s, 2H). ^{13}C NMR (101 MHz, DMSO-*D*6) δ 166.57, 166.40, 138.36, 138.28, 132.41, 128.29, 118.41, 113.67. ^{15}N NMR (41 MHz, DMSO-*D*6) δ -273.21.

HRMS (ESI) exact mass calculated for [M-H]- ($C_8H_6N^{15}NO$) requires m/z 146.04801, found m/z 146.02478.

4-(Amino-^{15}*N*)benzonitrile. Bromine (1.58 mL, 30.9 mmol) was added dropwise to 125 mL aqueous sodium hydroxide solution (1 M) at 0 °C. 4-cyanobenzamide-^{15}*N* (3.0 g, 20.6 mmol) was added and the

mixture stirred until fully dissolved. The solution was quickly heated to 70 °C with a pre-heated oil bath and stirred for 15 minutes before cooling to room temperature. The aqueous solution was extracted three times with dichloromethane, the combined organic phases washed with brine, dried with sodium sulfate and the solvent removed under reduced pressure. The resulting residue of 0.726 g (6.15 mmol, 30 %) 4-(amino-^{15}*N*)benzonitrile was used without further purification.

^{1}H NMR (400 MHz, CHLOROFORM-*D*) δ 7.50 – 7.36 (m, 2H), 6.76 – 6.61 (m, 2H), 4.18 (d, J = 56.0 Hz, 2H). ^{13}C NMR (101 MHz, CHLOROFORM-*D*) δ 150.60, 150.46, 133.92, 120.26, 114.56, 114.53, 100.28. ^{15}N NMR (41 MHz, CHLOROFORM-*D*) δ -315.30.

HRMS (EI) exact mass calculated for [M]+ ($C_7H_6N^{15}N$) requires m/z 119.053098, found m/z 119.13.

4,4',4''-(1,3,5-Triazine-2,4,6-triyl)trianiline-^{15}*N*. Under argon 4-(amino-^{15}*N*)benzonitrile (0.57 g, 4.82 mmol) was dissolved in 2 mL chloroform and trifluoromethanesulfonic acid (1.69 mL, 19.3 mmol) was added. The reaction mixture was stirred for 16 h at room temperature before 10 mL of water was carefully added and the mixture was neutralized with sodium hydroxide (1 M) to pH = 7. The precipitate was filtered off and washed with water, isopropanol, and dichloromethane. The solid was dried *in vacuo* to obtain 0.28 g (1.59 mmol, 49 %) 4,4',4''-(1,3,5-triazine-2,4,6-triyl)trianiline-^{15}*N.*

^{1}H NMR (400 MHz, DMSO-*D*6) δ 8.35 (d, J = 8.6 Hz, 2H), 6.69 (dd, J = 8.7, 1.7 Hz, 2H), 5.90 (d, J = 85.8 Hz, 2H). ^{13}C NMR (101 MHz, DMSO-*D*6) δ 169.57, 153.03, 152.90, 130.14, 122.91, 113.09, 39.52. ^{15}N NMR (41 MHz, DMSO-*D*6) δ -308.66.

HRMS (ESI) exact mass calculated for [M+H]+ ($C_{21}H_{18}N_3{}^{15}N_3$) requires m/z 358.159294, found m/z 358.15804.

COF Synthesis

Synthesis of ^{15}N Enriched TTI-COF

TTI-COF was synthesized following a literature procedure.[3] Into a 10 mL *Biotage* microwave vial, 4,4',4''-(1,3,5-triazine-2,4,6-triyl)trianiline-^{15}N (9 mg, 0.025 mmol), 4,4',4''-(1,3,5-triazine-2,4,6-triyl)trianiline (36 mg, 0.102 mmol) and 4,4',4''-(1,3,5-triazine-2,4,6-triyl)tribenzaldehyde (50 mg, 0.127) were placed. Mesitlyene (2.5 mL), 1,4-dioxane (2.5 mL), and 6M AcOH (0.125 mL) was added. The vial was capped and placed in an aluminum heating block that was preheated to 120°C. Under stirring at 500 rpm the mixture was kept at 120°C for 72h. After cooling to room temperature the solid

was isolated by filtration, washed with acetone, isopropanol, and methanol before subjecting it to a Soxhlet extraction with MeOH for 24h. The MeOH soaked solid was then activated by scCO2 drying to obtain TTI-COF (67.6 mg, 77 %).

Synthesis of ^{15}N Enriched rTTI-COF

rTTI-COF was synthesized following a literature procedure.[4] ^{15}N enriched TTI-COF (90 mg, 0.203 mmol) was suspended in mesitylene (6 mL) and 1,4-dioxane (3 mL). Formic acid (97%, 59.0 µL) was added and the suspension was heated at 120°C for 48 h. The solid was isolated by filtration, washed with methanol and subjected to a Soxhlet extraction with MeOH for 24h. The MeOH soaked solid was then activated by scCO2 drying to obtain TTI-COF (84 mg, 93 %).

Synthesis of ^{15}N Enriched TTT-COF

TTT-COF was synthesized following a literature procedure.[5] ^{15}N enriched TTI-COF (80 mg, 0.203 mmol) was thoroughly mixed with sulfur (1.2 g, 37.4 mmol) in a mortar and the homogeneous mixture was transferred to an aluminum oxide boat in a horizontal tubular furnace. Under constant argon flow, the tube was purged at 60 °C for 1 h followed by a temperature increase to 155 °C (1 K min^{-1} heating rate) for 3 h and a further increase to 350 °C (1.5 K min^{-1} heating rate) for another 3 h. After cooling to room temperature, the solid was subjected to Soxhlet extractions with CS_2 and methanol (24 h each). The MeOH soaked solid was then activated by scCO2 drying to obtain TTT-COF (72.2 mg, 80 %).

Synthesis of ^{15}N Enriched TT-Imide-COF

TT-Imide-COF was synthesized following a literature procedure.[6] A Schlenk tube was charged with the precursor molecules pyromellitic dianhydride (PMDA, 32.7 mg, 0.15 mmol) and ^{15}N enriched 2,4,6-tris(4-aminophenyl)-1,3,5-triazine (TT, 35.4 mg, 0.1 mmol) together with a mixture of 0.5 ml N-methyl-2-pyrrolidone, 0.5 ml mesitylene and 0.05 ml isoquinoline. The reaction mixture was degassed by four freeze-pump-thaw cycles and subsequently heated to 180 °C for 5 days. A yellow precipitate was formed, isolated by filtration and subjecting it to Soxhlet extractions with THF and methanol (24 h each). The MeOH soaked solid was then activated by scCO2 drying to obtain TT-Imide-COF (40.8 mg, 65 %).

Exposure of COFs to NO

The COFs were exposed to nitric oxide via the NO adsorption measurements on the Quantachrom Autosorb IQ3 system. All analytics on the post-NO materials were performed after the performance of

all NO sorption experiments, including seven succeeding NO adsorption/desorption isotherms at 298 K and three isotherms at 298 K, 288 K and 273 K, respectively, to ensure full reaction of the frameworks with the gas.

NO release experiments

The NO release experiment was conducted by suspending 10 mg rTTI-COF-*NO* in 5 ml 0.1 M PBS buffer solution. The temperature was kept constant at 37 °C during the observation period and the mixture was shaken permanently to avoid precipitation of the COF. To determine the concentration of NO in the mixture, a Griess reagent kit for nitrite determination (G-7921) produced by *Probes* has been used. The conversion of the released nitric oxide to nitrite appears *in situ* by atmospheric oxygen:

$$2\,NO + O_2 \longrightarrow 2\,NO_2$$

$$2\,NO + 2\,NO_2 \longrightarrow 2\,N_2O_3$$

$$2\,N_2O_3 + 2\,H_2O \longrightarrow 4\,NO_2^- + 4\,H^+$$

The formed nitrite is detected by using the Griess reagent through the formation of an azo-dye following the reaction:

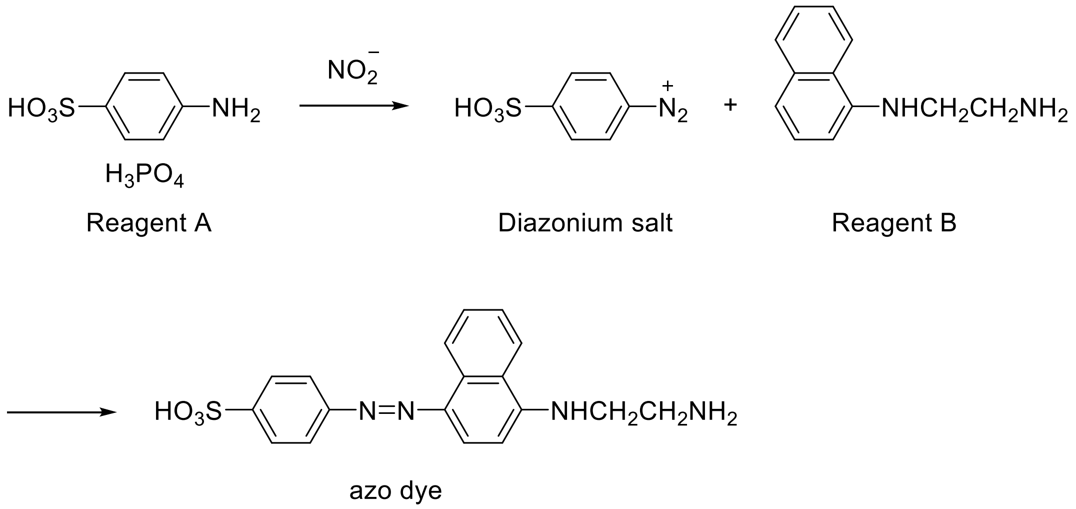

For each measurement, 500 µl sample were taken and centrifuged. 300 µl of the supernatant were mixed with 100 µl Griess reagent and diluted with 2.6 ml water. After 30 min dwelling time, the nitrite concentration was measured by UV/VIS spectroscopy using a serial dilution as reference. The remaining 200 µl supernatant together with the centrifuged COF were subsequently combined with the initial NO-release mixture again. Unavoidable volume changes through the sample withdrawal during the long-term experiment (300 µl) have been considered in the NO release calculations.

Supplementary Data

Supplementary PXRD Data

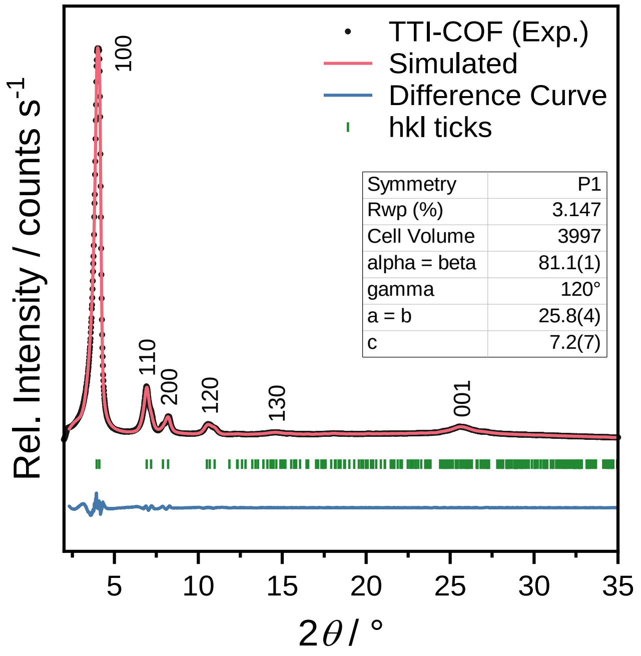

Figure S1. Experimental PXRD pattern of TTI-COF (black) together with the Pawley fit (red), calculated reflection positions, (green) and difference curves (blue).

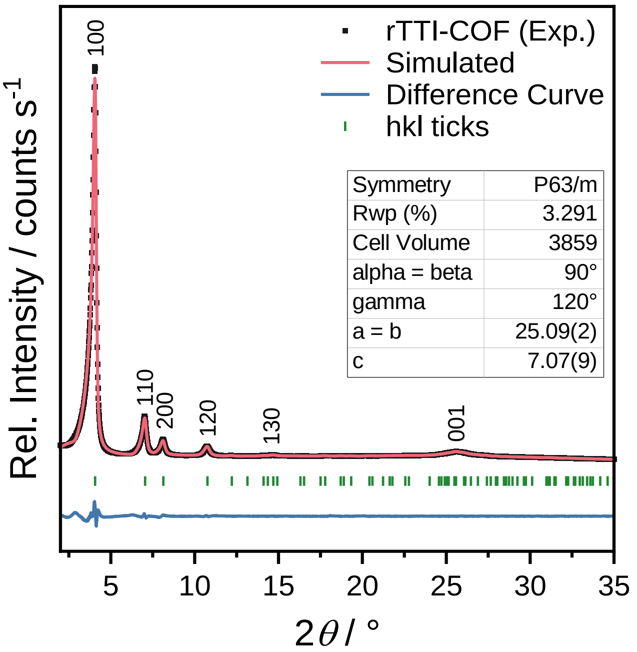

Figure S2. Experimental PXRD pattern of rTTI-COF (black) together with the Pawley fit (red), calculated reflection positions, (green) and difference curves (blue).

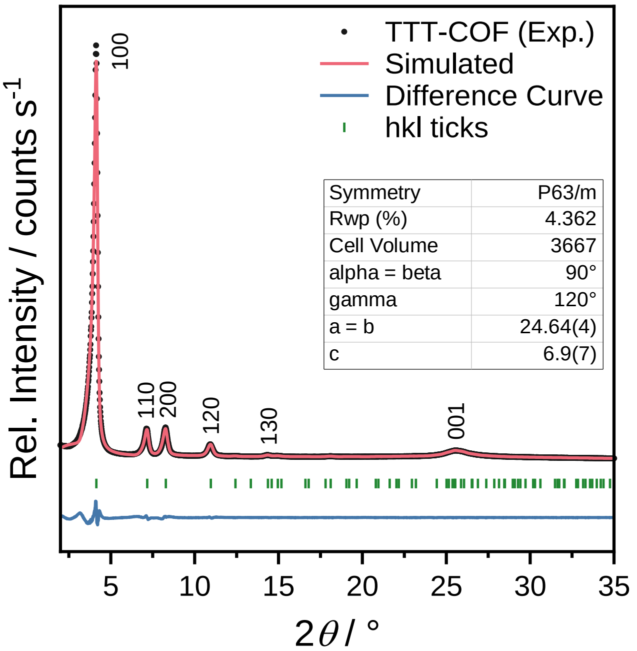

Figure S3. Experimental PXRD pattern of TTT-COF (black) together with the Pawley fit (red), calculated reflection positions, (green) and difference curves (blue).

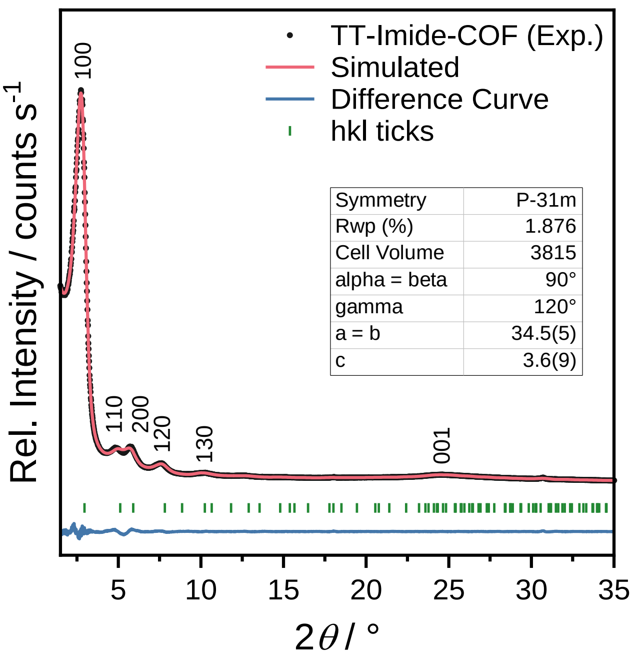

Figure S4. Experimental PXRD pattern of TT-Imide-COF (black) together with the Pawley fit (red), calculated reflection positions (green), and difference curves (blue).

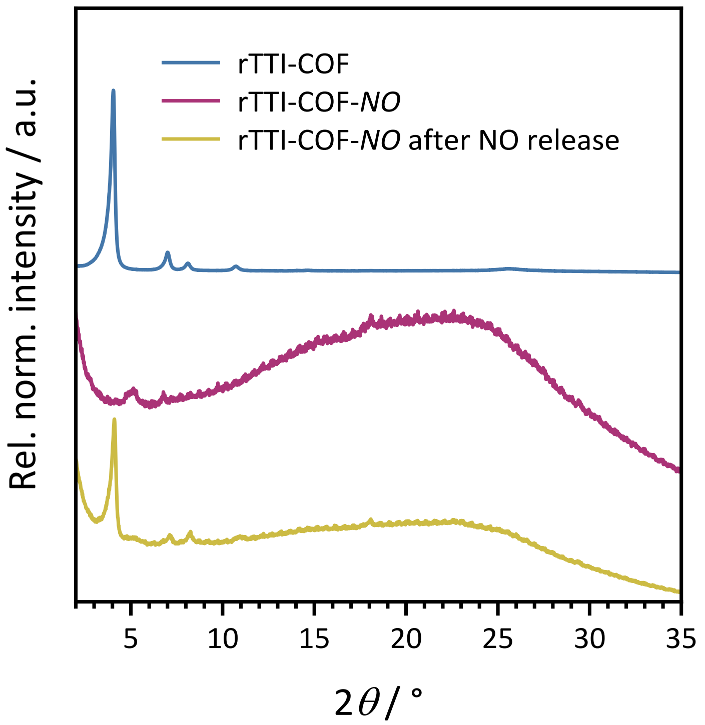

Figure S5. Comparison of experimental PXRD patterns of rTTI-COF (blue), rTTI-COF-*NO*, and rTTI-COF-*NO* after NO release.

Supplementary FT-IR Spectra

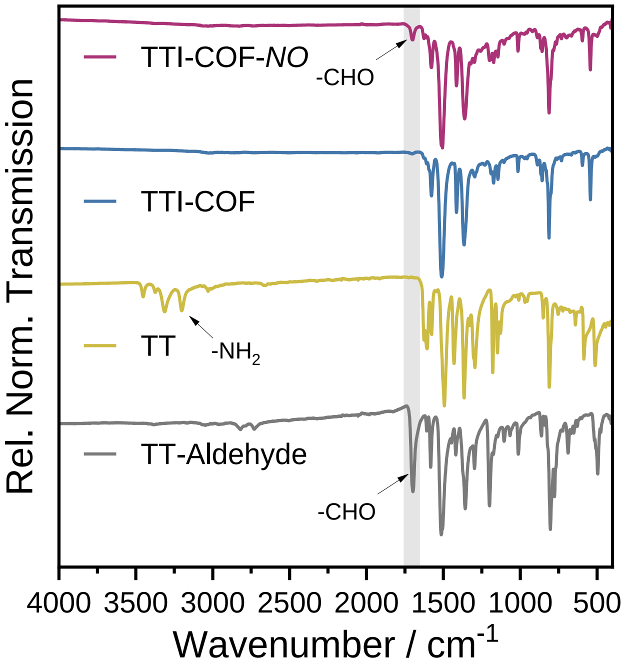

Figure S6. FT-IR spectra of TTI-COF before (blue) and after NO sorption experiments (red) together with FT-IR spectra of the precursor molecules TT-Aldehyde (black) and TT (yellow). The absence of amine vibrational bands at 3367 cm^{-1} and aldehyde vibrational bands at 1698 cm^{-1} in the COF spectrum indicate complete imine formation. After NO treatment the aldehyde vibrational band at 1698 cm^{-1} of the precursor TT-Aldehyde appears again, indicating imine bond breaking.

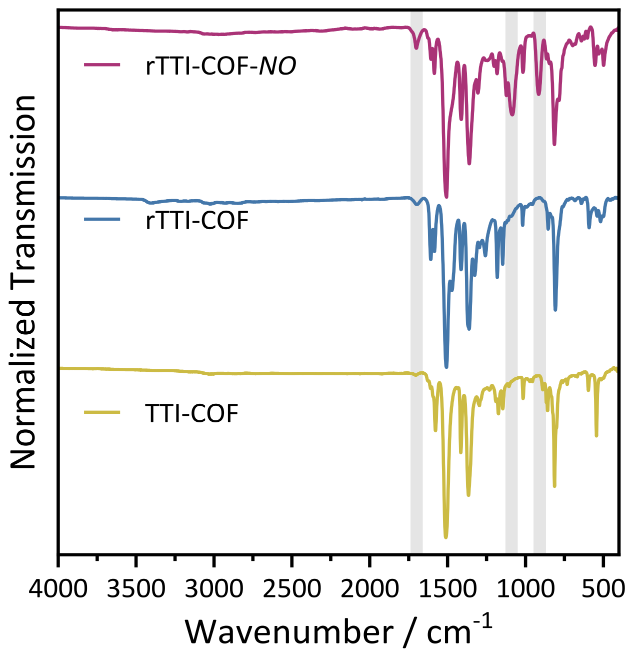

Figure S7. FT-IR spectra of rTTI-COF before (blue) and after NO adsorption experiments (red) together with the FT-IR spectrum of the precursor TTI-COF (yellow). After NO treatment three additional vibrational bands at 1700 cm^{-1}, 1084 cm^{-1}, and 916 cm^{-1} appear, indicating the formation of the NONOate.

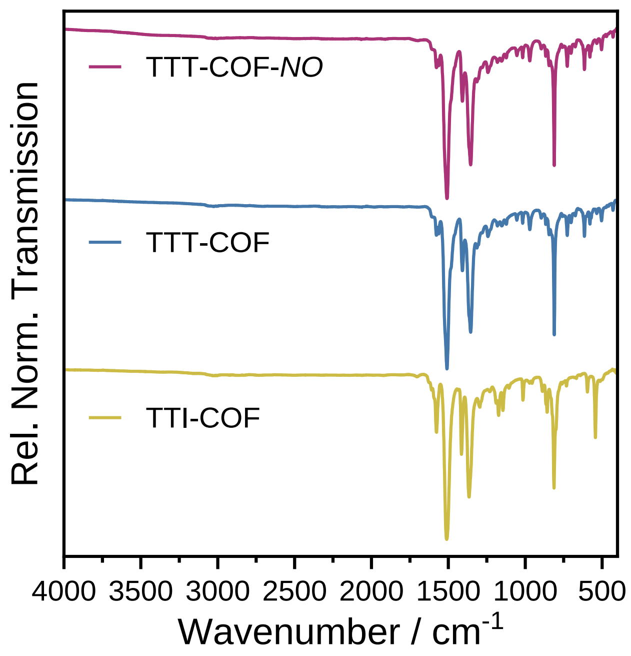

Figure S8. FT-IR spectra of TTT-COF before (blue) and after NO adsorption experiments (red) together with the FT-IR spectrum of the precursor TTI-COF (yellow). No changes can be observed in the spectrum of TTT-COF after NO treatment, indicating inertness of the system against NO.

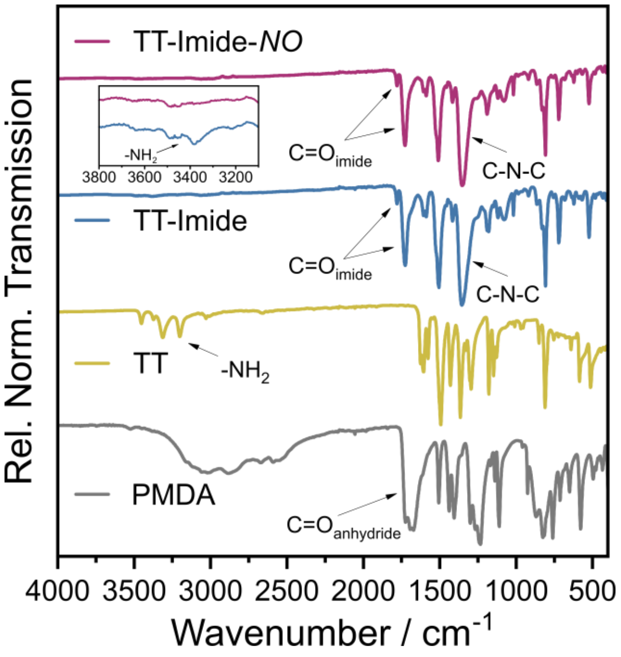

Figure S9. FT-IR spectra of TT-Imide-COF before (blue) and after NO adsorption experiments (red) together with FT-IR spectra of the precursor molecules PMDA (black) and TT (yellow). The absence of amine vibrational bands at 3367 cm^{-1} and anhydride vibrational bands at 1700 cm^{-1} in the COFs spectrum indicate complete imide formation. No strong changes can be observed in the spectrum after NO treatment, indicating strong inertness of the system towards NO. Inset shows the disappearance of faint amine vibrational bands at 3367 cm^{-1} after the NO-treatment, which are in good agreement to the findings of by ^{15}N CP-MAS ssNMR.

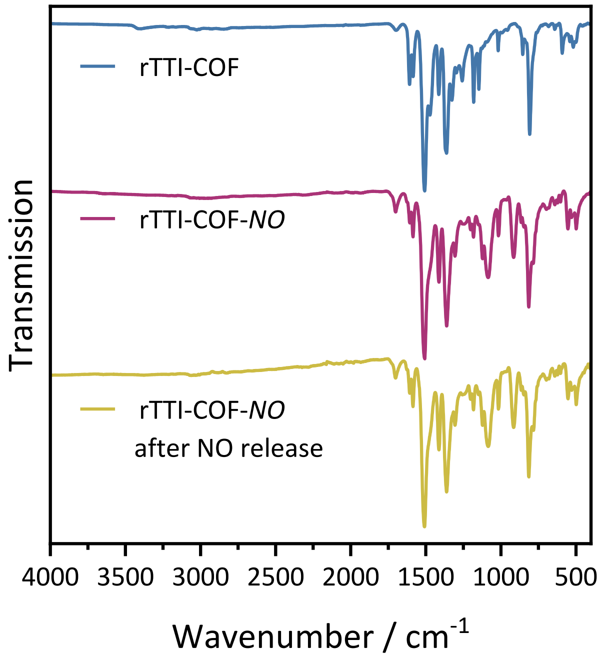

Figure S10. FT-IR spectra of rTTI-COF before (blue) and after NO adsorption experiments (red) as well as after the NO release (yellow), which show no significant changes.

Supplementary NMR Spectra

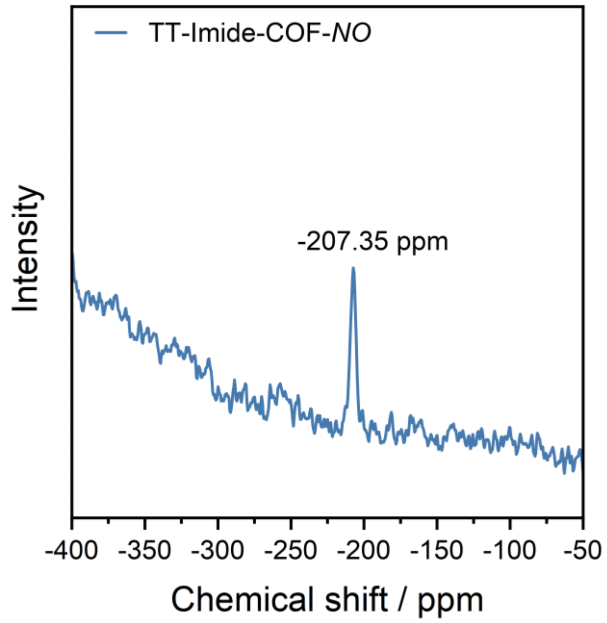

Figure S11. ^{15}N direct excitation NMR spectrum of TT-Imide COF showing one signal corresponding to the imide nitrogen at -207.35 ppm.

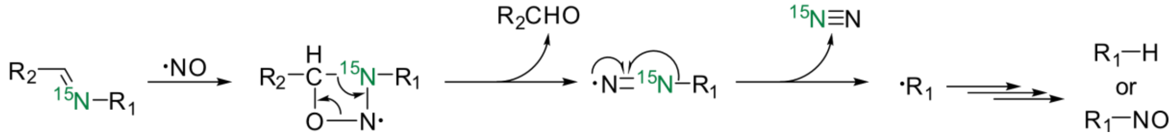

Scheme S2. Mechanism of imine linkage degradation by a [2+2] cycloaddition of NO to the imine bond, proposed by Hrabie et al.[7]

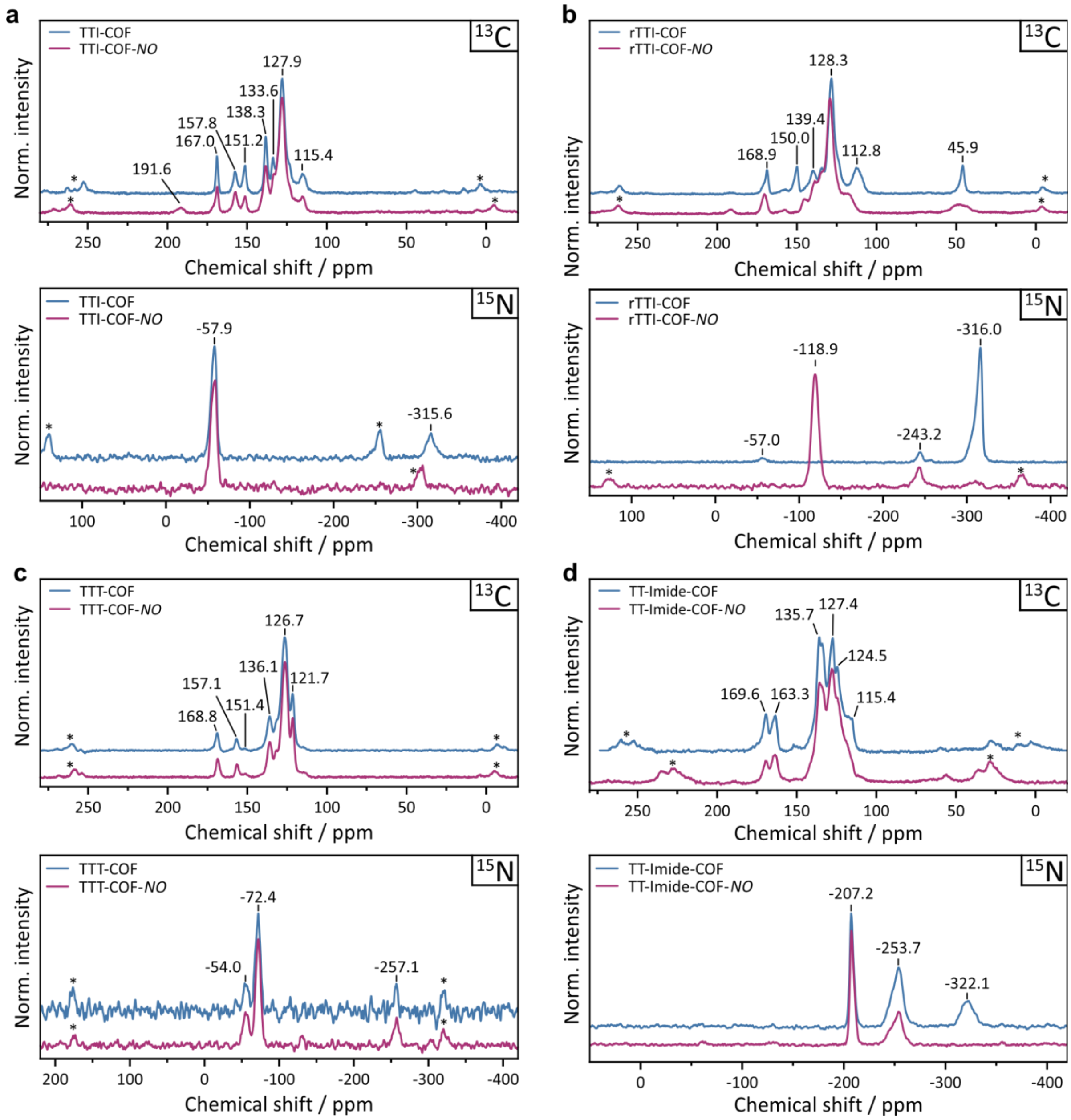

Figure S12. Direct comparison of ^{13}C CP-MAS (top) and ^{15}N CP-MAS ssNMR (bottom) of (a) TTI-COF, (b) rTTI-COF), (c) TTT-COF and (d) TT-Imide-COF before and after NO exposure.

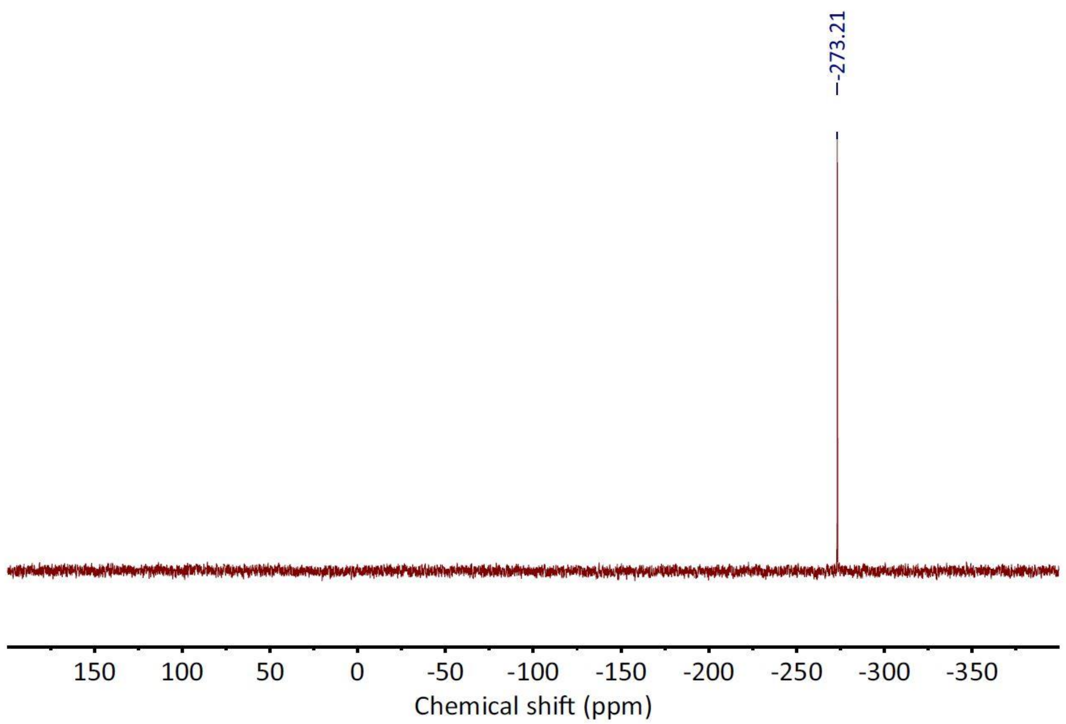

Figure S13. ^{15}N NMR of **4-cyanobenzamide-^{15}*N*** in DMSO-*D6*.

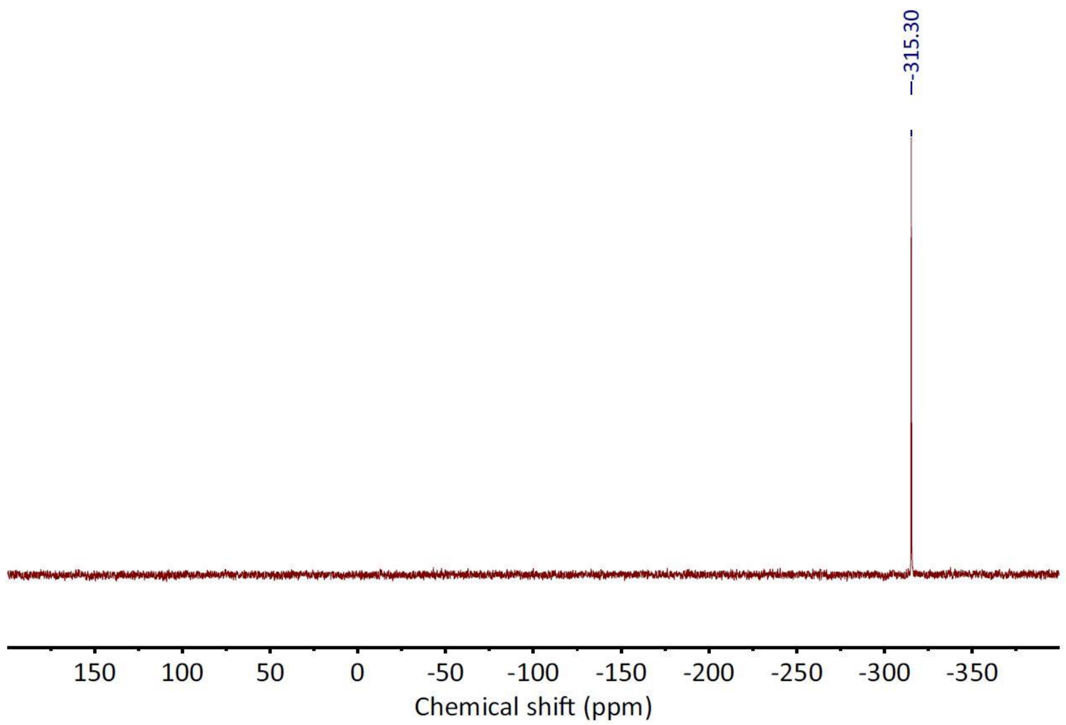

Figure S14. ^{15}N NMR of **4-(amino-^{15}*N*)benzonitrile** in $CDCl_3$.

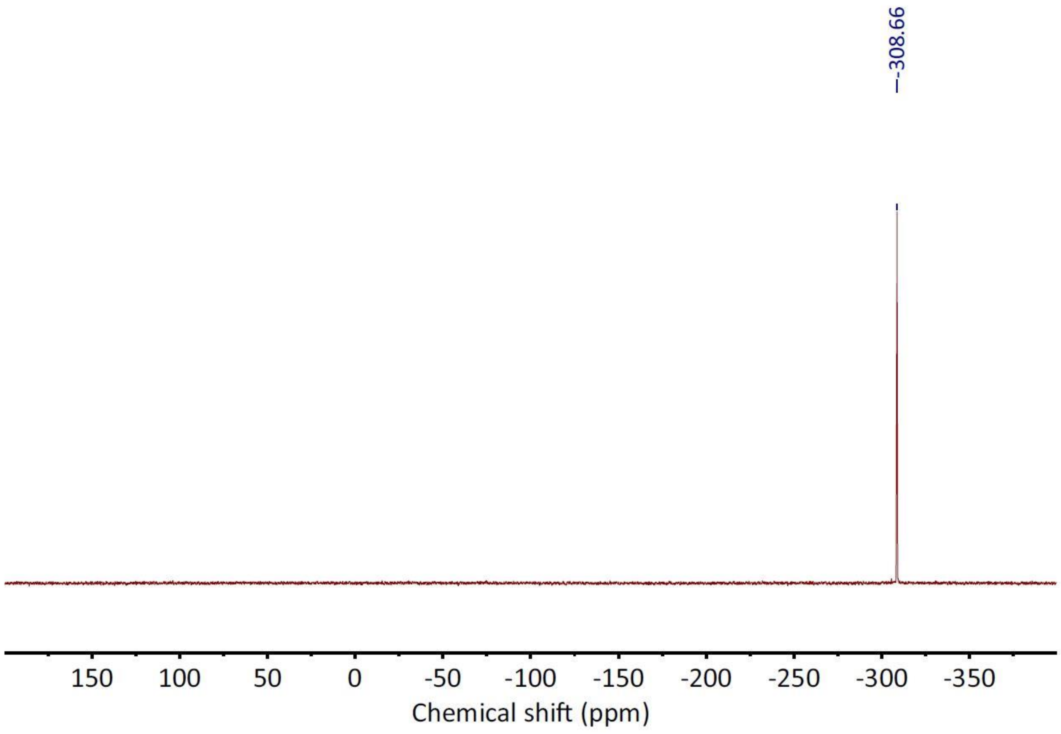

Figure S15. ^{15}N NMR of **4,4',4''-(1,3,5-triazine-2,4,6-triyl)trianiline-^{15}*N*** in DMSO-*D*6.

Supplementary TEM Images

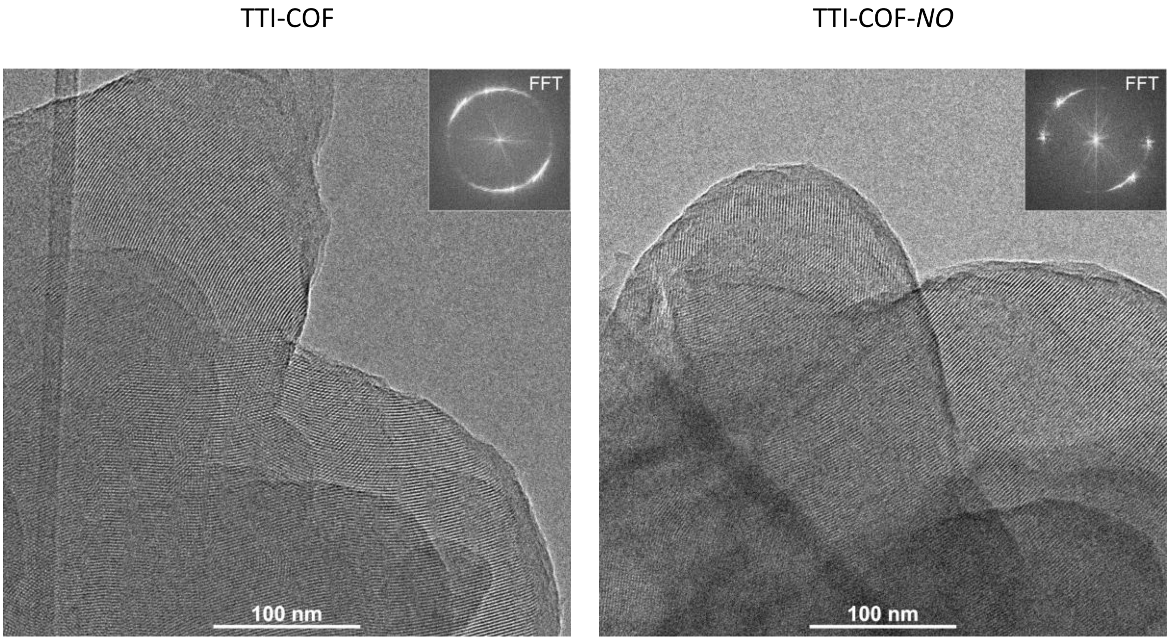

Figure S16. TEM images of TTI-COF before (left) and after NO sorption measurements (right) showing retention of the crystalline structure.

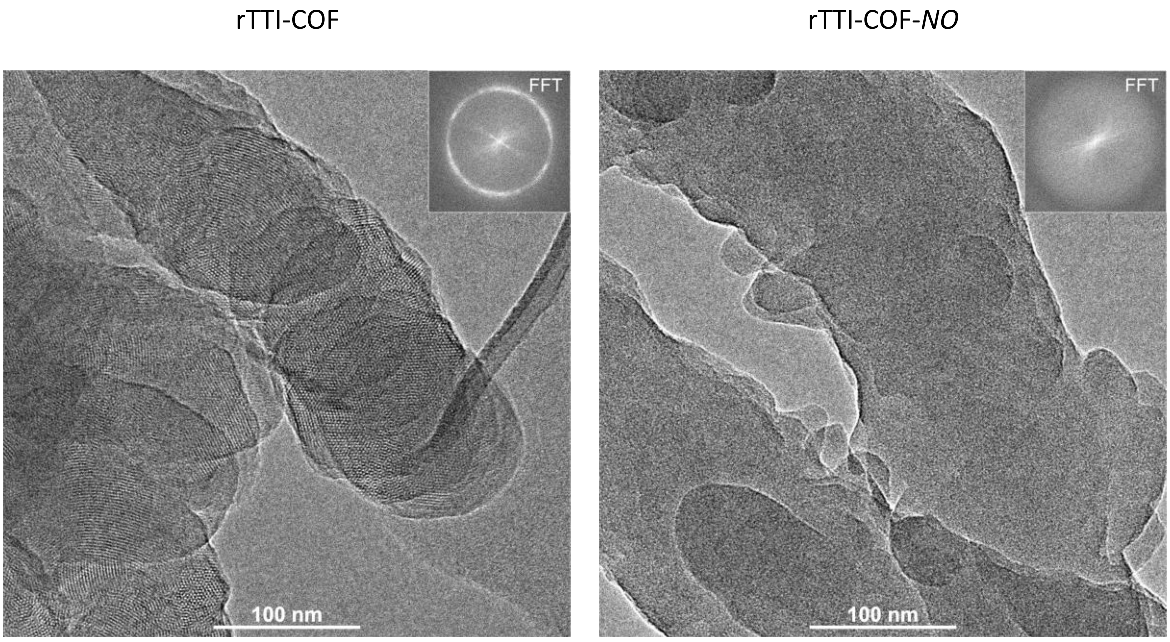

Figure S17. TEM images of rTTI-COF before (left) and after NO sorption measurements (right) revealing a collapse of the crystalline structure.

TTT-COF TTT-COF-*NO*

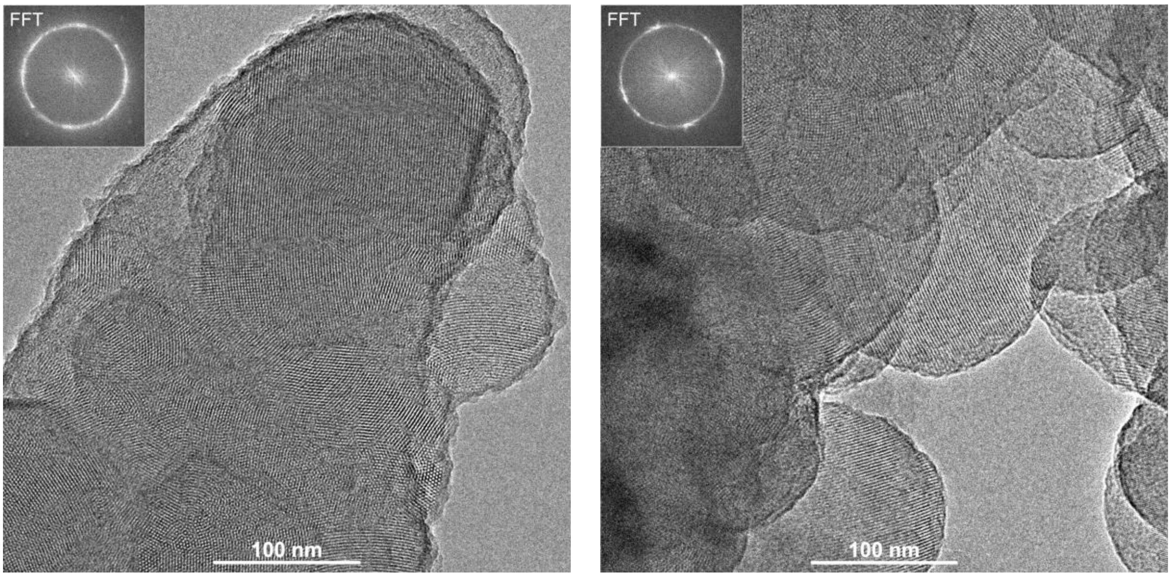

Figure S18. TEM images of TTT-COF before (left) and after NO sorption measurements (right) showing retention of the crystalline structure.

TT-Imide-COF TT-Imide-COF-*NO*

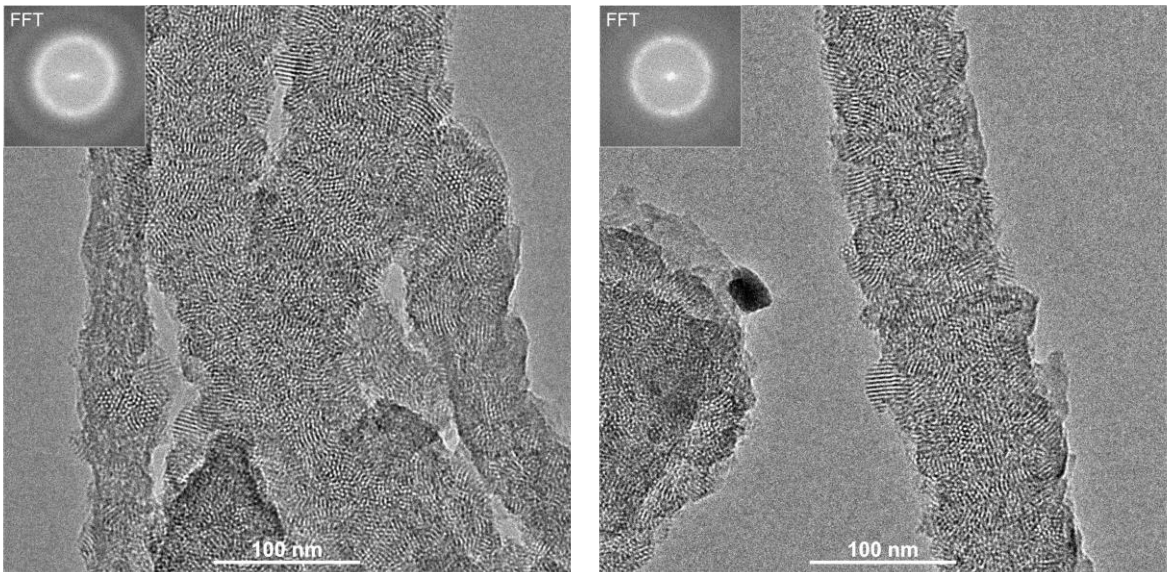

Figure S19. TEM images of TT-Imide-COF before (left) and after NO sorption measurements (right) showing retention of the crystalline structure.

Supplementary UV/VIS Spectra

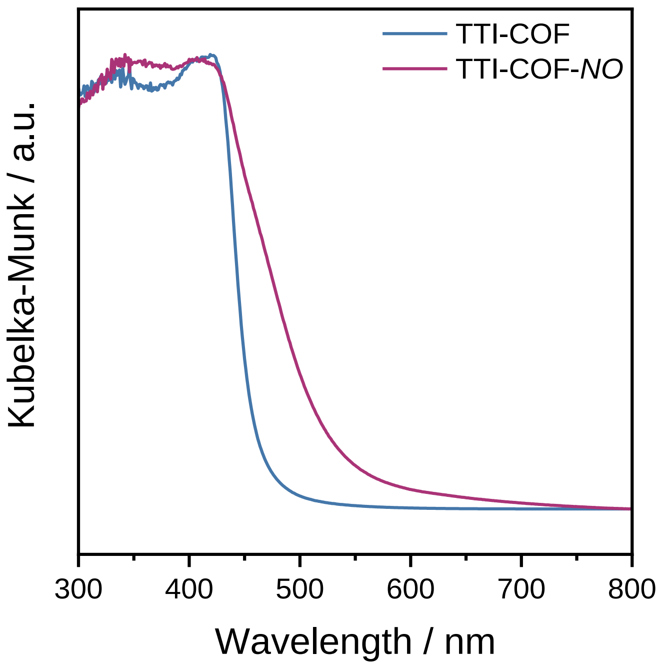

Figure S20. Diffuse reflectance spectra of TTI-COF before (blue) and after NO sorption experiments (red) revealing a shift of the absorption towards higher wavelengths, along with broadening/tailing of the absorption edge.

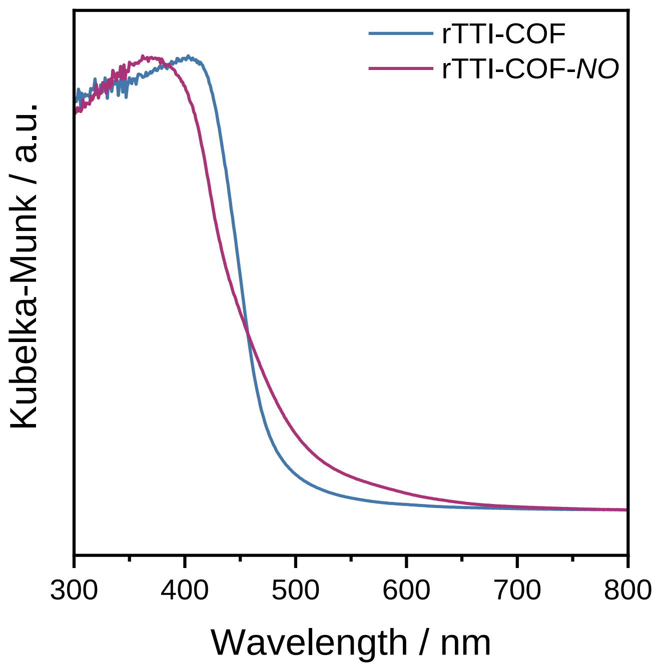

Figure S21. Diffuse reflectance spectra of rTTI-COF before (blue) and after NO sorption experiments (red) revealing a slight blueshift in the absorption edge, along with broadening/tailing.

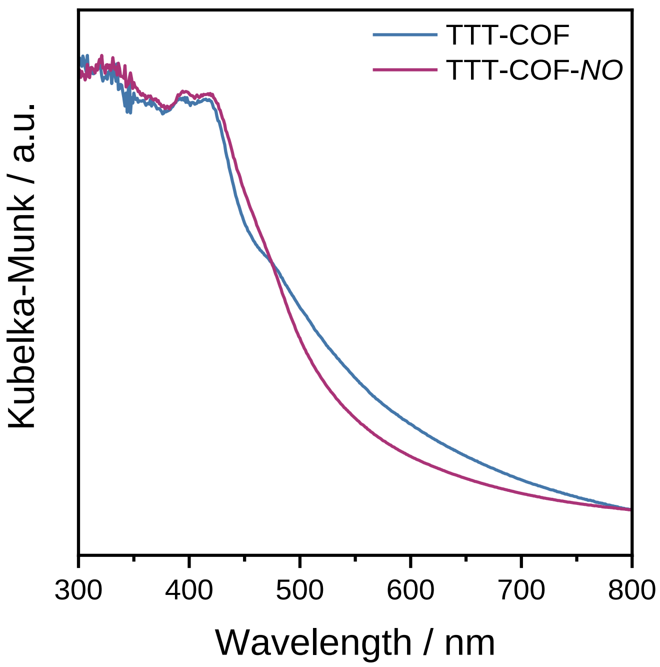

Figure S22. Diffuse reflectance spectra of TTT-COF before (blue) and after NO sorption experiments (red) showing slight changes in the structure of the absorption edge.

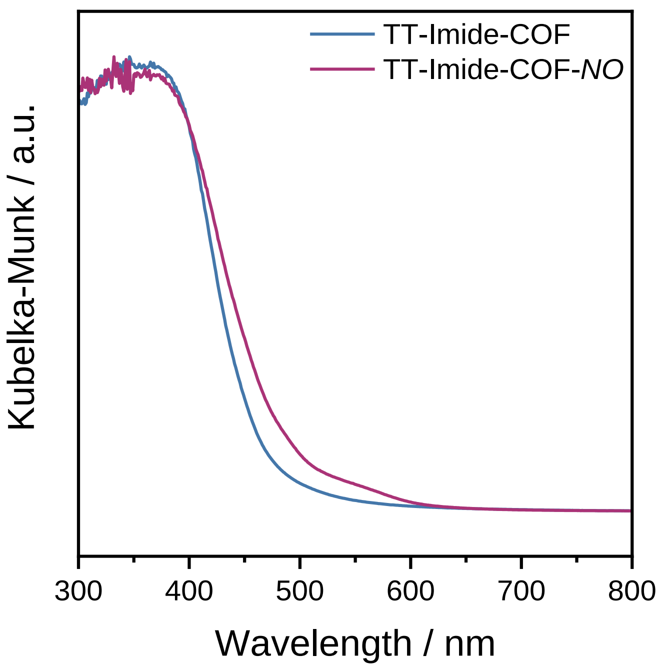

Figure S23. Diffuse reflectance spectra of TT-Imide-COF before (blue) and after NO sorption experiments (red), suggesting that the optical properties are largely retained.

Supplementary Sorption Data

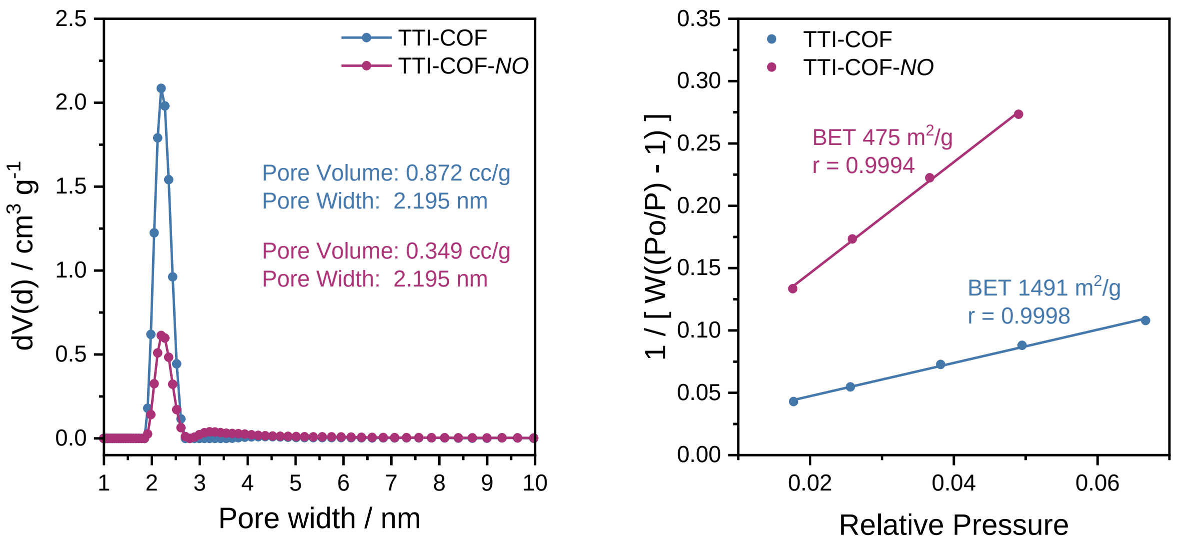

Figure S24. Calculated pore size distributions (left) and BET plots (right) of TTI-COF before (blue) and after NO sorption experiments (red), revealing a significant decrease in the surface area and pore volume after NO treatment.

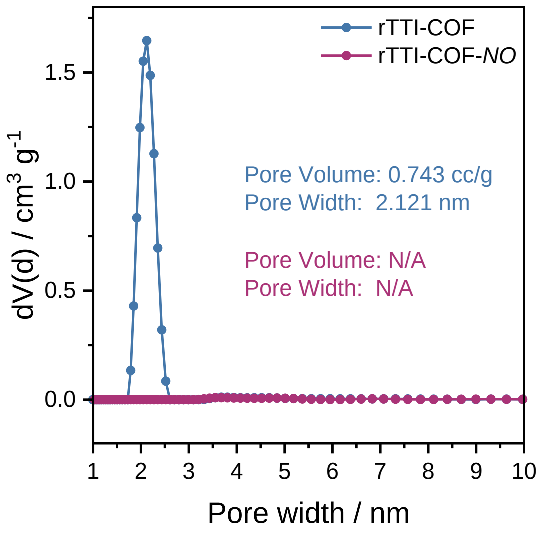

Figure S25. Calculated pore size distributions of rTTI-COF before (blue) and after NO sorption experiments (red), revealing a complete loss of structural porosity after NO treatment.

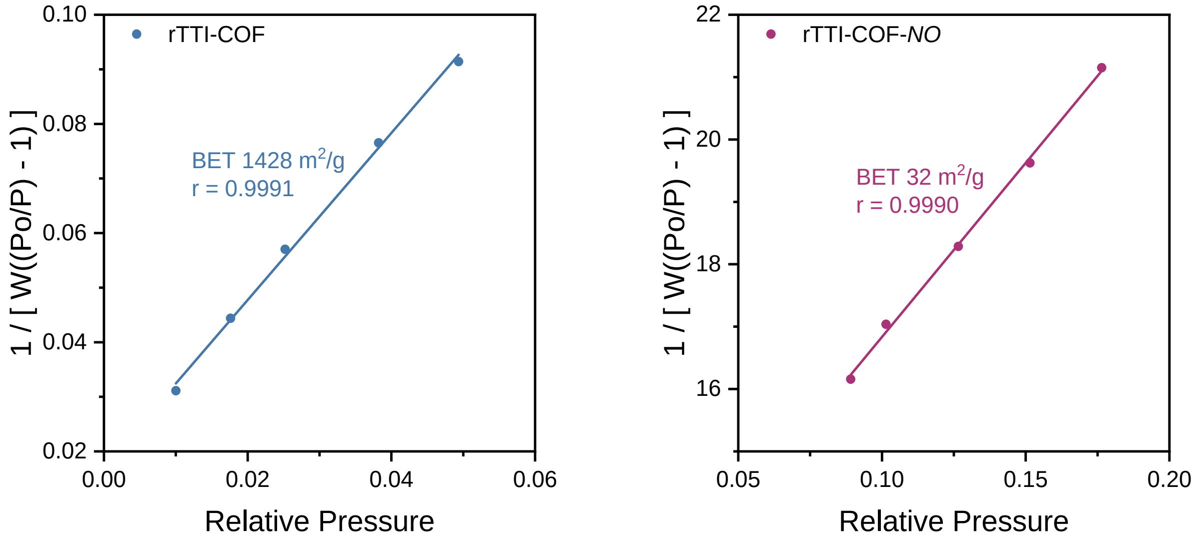

Figure S26. BET plots of rTTI-COF before (blue) and after NO sorption experiments (red), revealing an essentially complete loss of surface area after NO treatment.

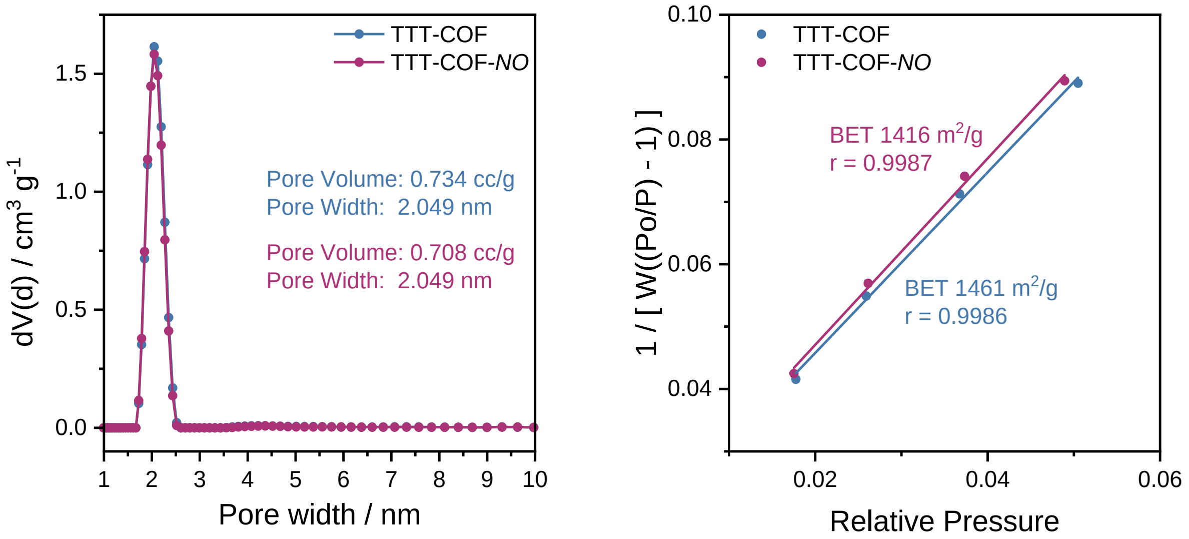

Figure S27. Calculated pore size distributions (left) and BET plots (right) of TTT-COF before (blue) and after NO sorption experiments (red), showing retention of its surface area and pore volume upon NO treatment indicating a strong robustness of the framework against NO.

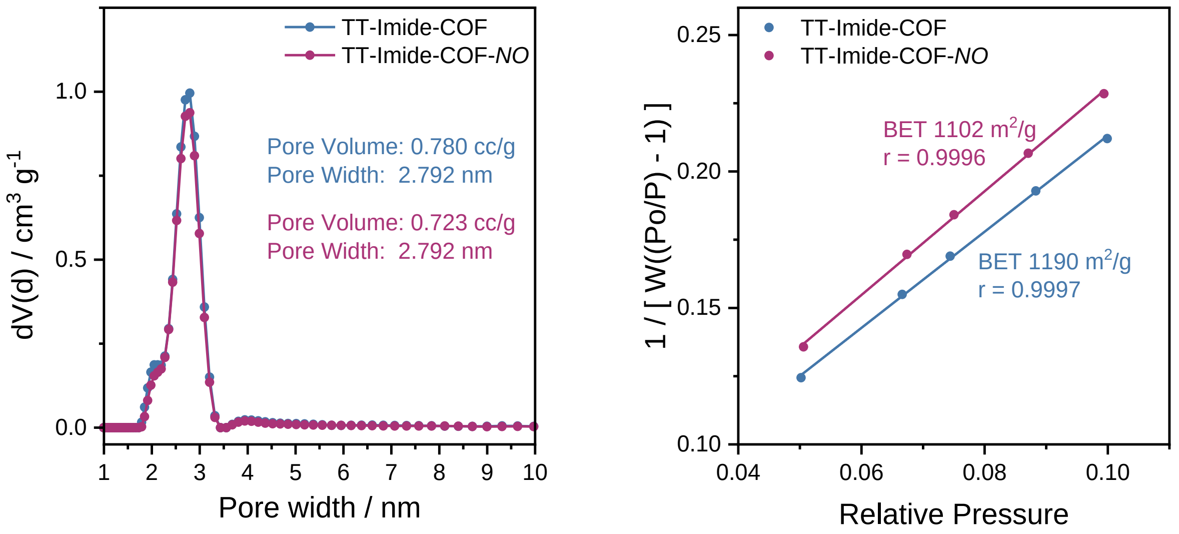

Figure S28. Calculated pore size distributions (left) and BET plots (right) of TT-Imide-COF before (blue) and after NO sorption experiments (red), showing retention of its surface area and pore volume upon NO treatment, indicating that the framework is robust against NO.

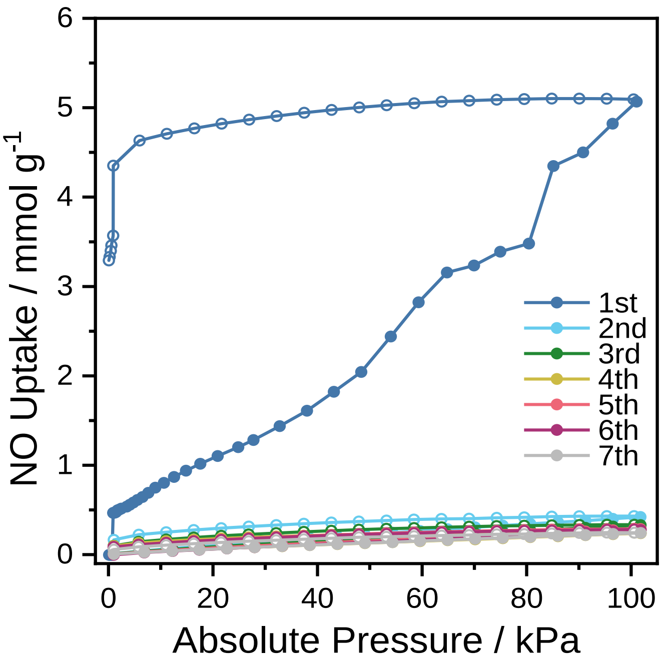

Figure S29. First seven NO adsorption and desorption isotherms of TTI-COF showing an unusual increased NO uptake within the first cycle. After the first cycle, the total uptake decreases until it stabilizes after three cycles.

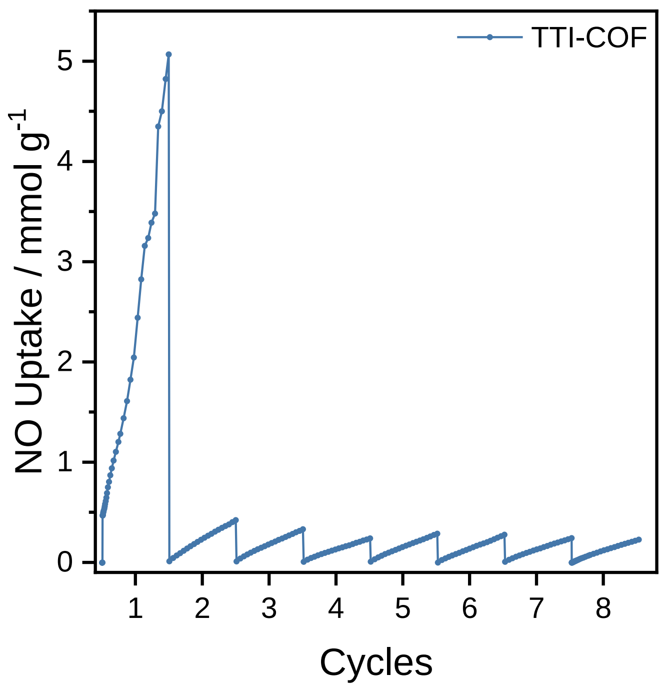

Figure S30. NO adsorption isotherms of TTI-COF plotted in a row, demonstrating the stabilization in NO uptake behavior after two to three cycles.

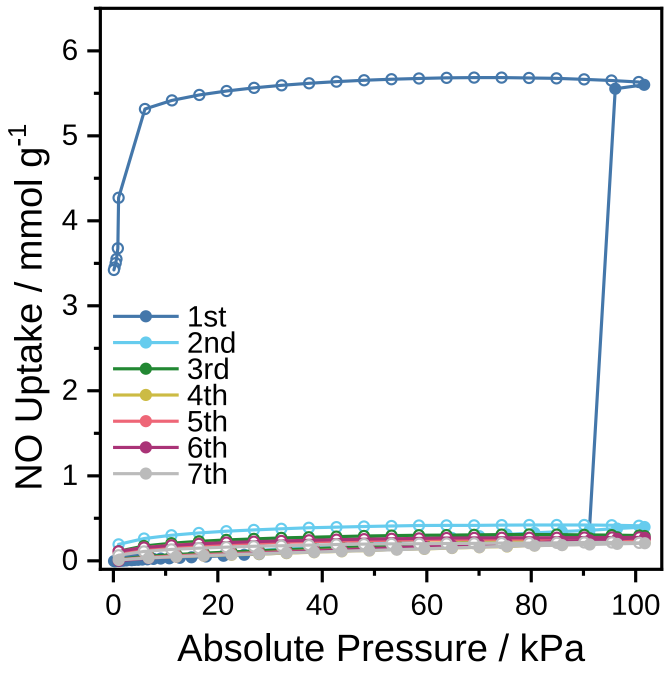

Figure S31. First seven NO adsorption and desorption isotherms of rTTI-COF showing an unusual increased NO uptake within the first cycle. After the first cycle, the total uptake decreases until it stabilizes after three cycles.

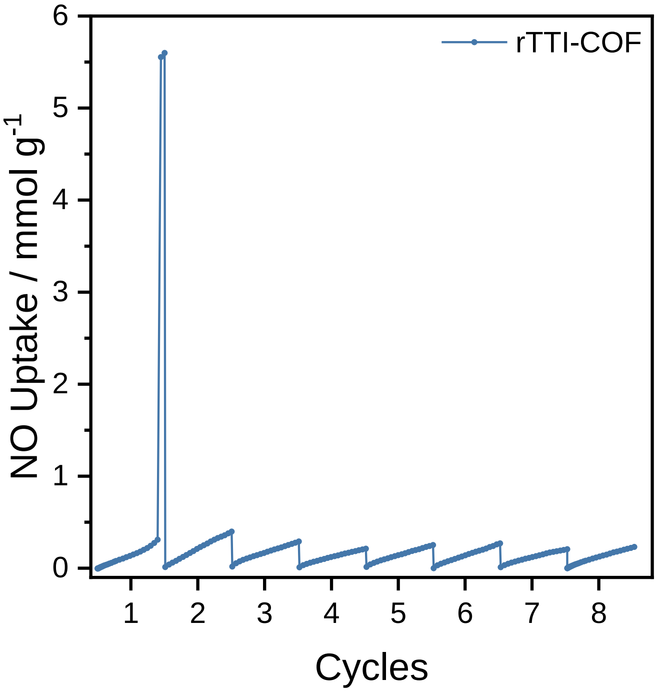

Figure S32. NO adsorption isotherms of rTTI-COF plotted in a row demonstrating the stabilization of NO uptake behavior after two to three cycles.

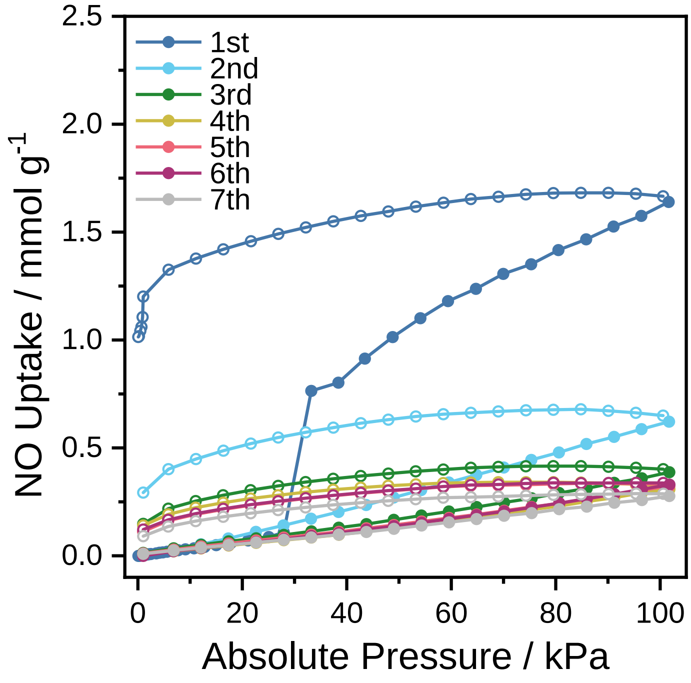

Figure S33. First seven NO adsorption and desorption isotherms of TTT-COF showing an unusual increased NO uptake within the first two cycles. Over the first and second cycle, the total uptake decreases until it stabilizes after three cycles.

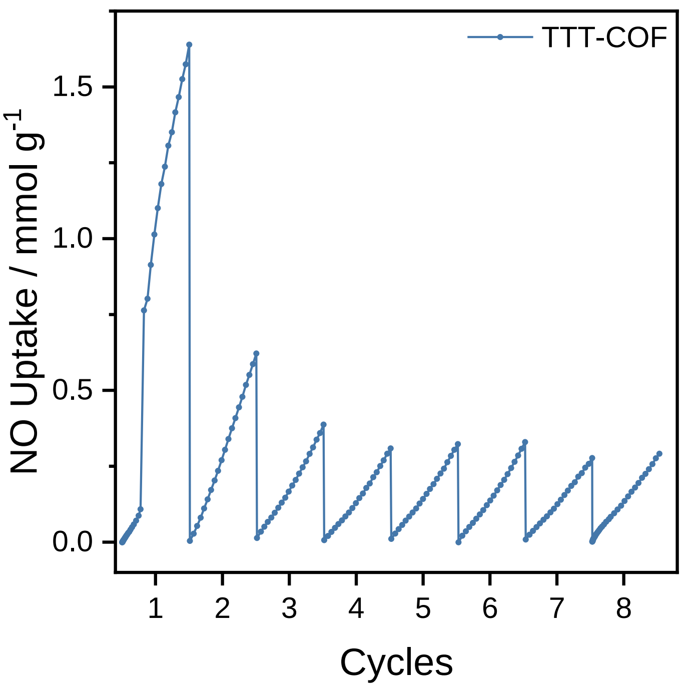

Figure S34. NO adsorption isotherms of TTT-COF plotted in a row demonstrating stabilization of the NO uptake behavior after three cycles.

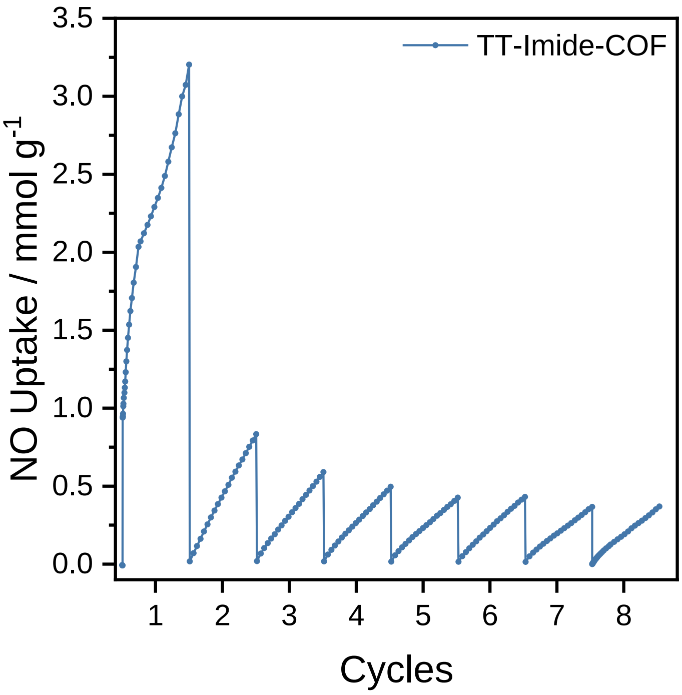

Figure S35. NO adsorption isotherms of TT-Imide-COF plotted in a row, demonstrating stabilization of the NO uptake behavior after two to four cycles.

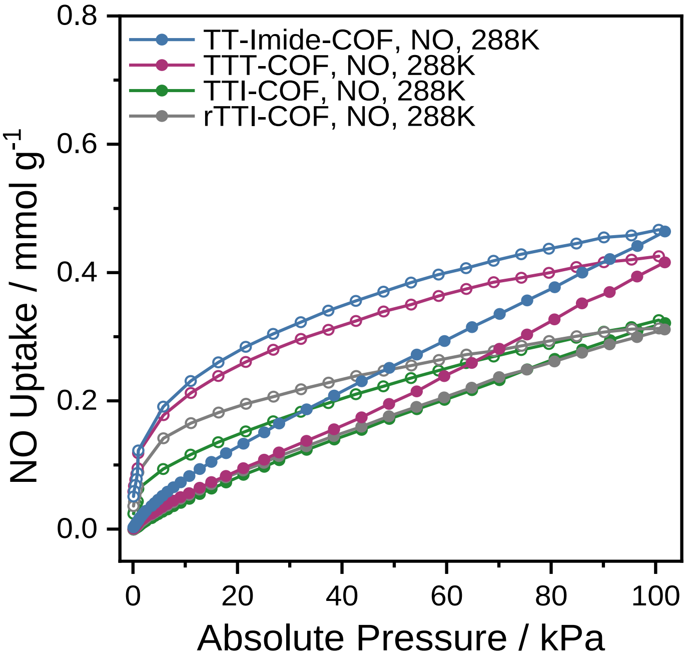

Figure S36. NO adsorption and desorption isotherms of TTI-, rTTI-, TTT-, and TT-Imide-COF at 288 K, after the cycling experiments. The reversibility of the isotherms indicates a process that can be described by physisorption.

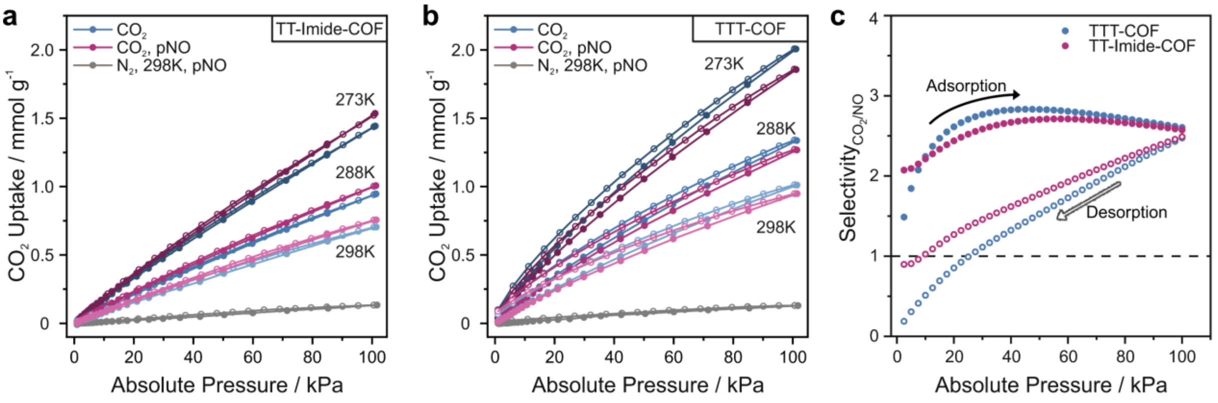

Figure S37. CO_2 isotherms of (a) TT-Imide-COF and (b) TTT-COF before (blue) and after NO exposure (red). Filled circles represent adsorption and empty circles desorption isotherms. (c) IAST selectivity of TTT-COF-NO and TT-Imide-COF-NO for a binary CO_2/NO (50/50) gas mixture during adsorption (filled circles) and desorption (empty circles). For a binary CO_2/NO (50/50) gas mixture, we found a selectivity towards CO_2 over the whole pressure range during adsorption up to 100 kPa for both COFs. The selectivities first increase until around 50 kPa before slightly declining to an almost identical value of 2.5 at 100 kPa. However, applying IAST to the desorption instead of the adsorption branch, in both COFs, the selectivity changes in favor of NO for low pressures below 30 kPa. In our opinion, this practice is a better representation of the thermodynamic equilibrium state due to the unusual and broad hysteresis of the pure NO isotherm.

Table S1. Calculated heats of adsorption, CO_2 and NO adsorption capacities and IAST selectivities for TT-Imide-COF-NO and TTT-COF-NO.

	Initial Q_{st} CO_2 (kJ mol^{-1})	CO_2 capacity (mmol g^{-1})		Selectivity[a] CO_2/N_2 (IAST)	Initial Q_{st} NO (kJ mol^{-1})	NO capacity (mmol g^{-1})		Selectivity[b] NO/N_2 (IAST)
		273K	298K			273K	298K	
TT-Imide-COF-*NO*	26.6-20.5	1.86	0.944	9.22	35.7-16.9	0.623	0.367	5.55
TTT-COF-*NO*	23.8-20.8	1.54	0.755	6.44	21.6-17.0	0.562	0.290	3.95

[a] Selectivity was calculated for 15/85 gas mixtures at 1 bar and 298 K for CO_2/N_2

[b] Selectivity was calculated for 3/97 gas mixtures at 1 bar and 298 K for NO/N_2

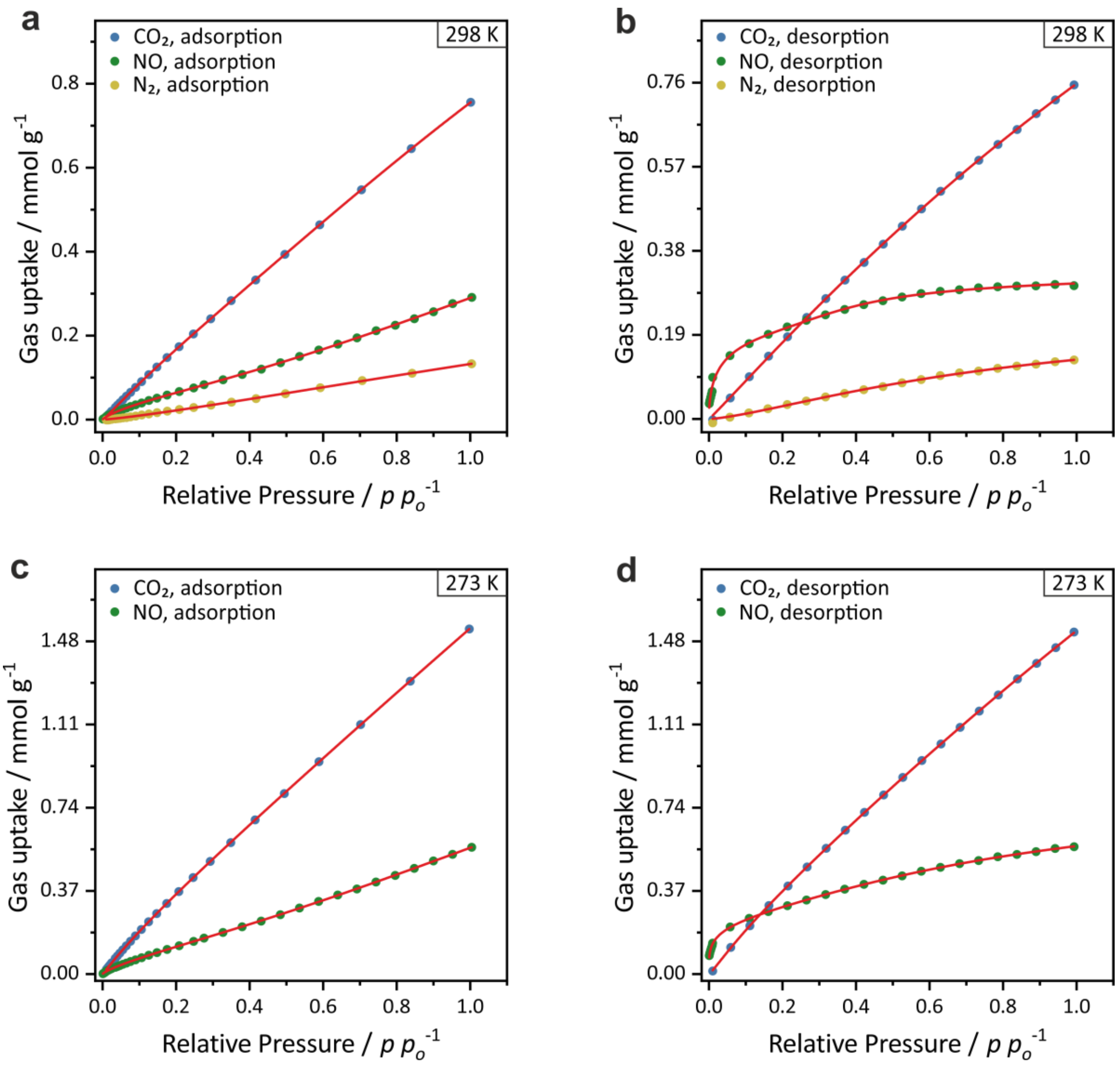

Figure S38. Fitting curves of **a** the adsorption isotherms at 298 K, **b** the desorption isotherms at 298 K, **c** the adsorption isotherms at 273 K and **d** the desorption isotherms at 273 K of TTT-COF.

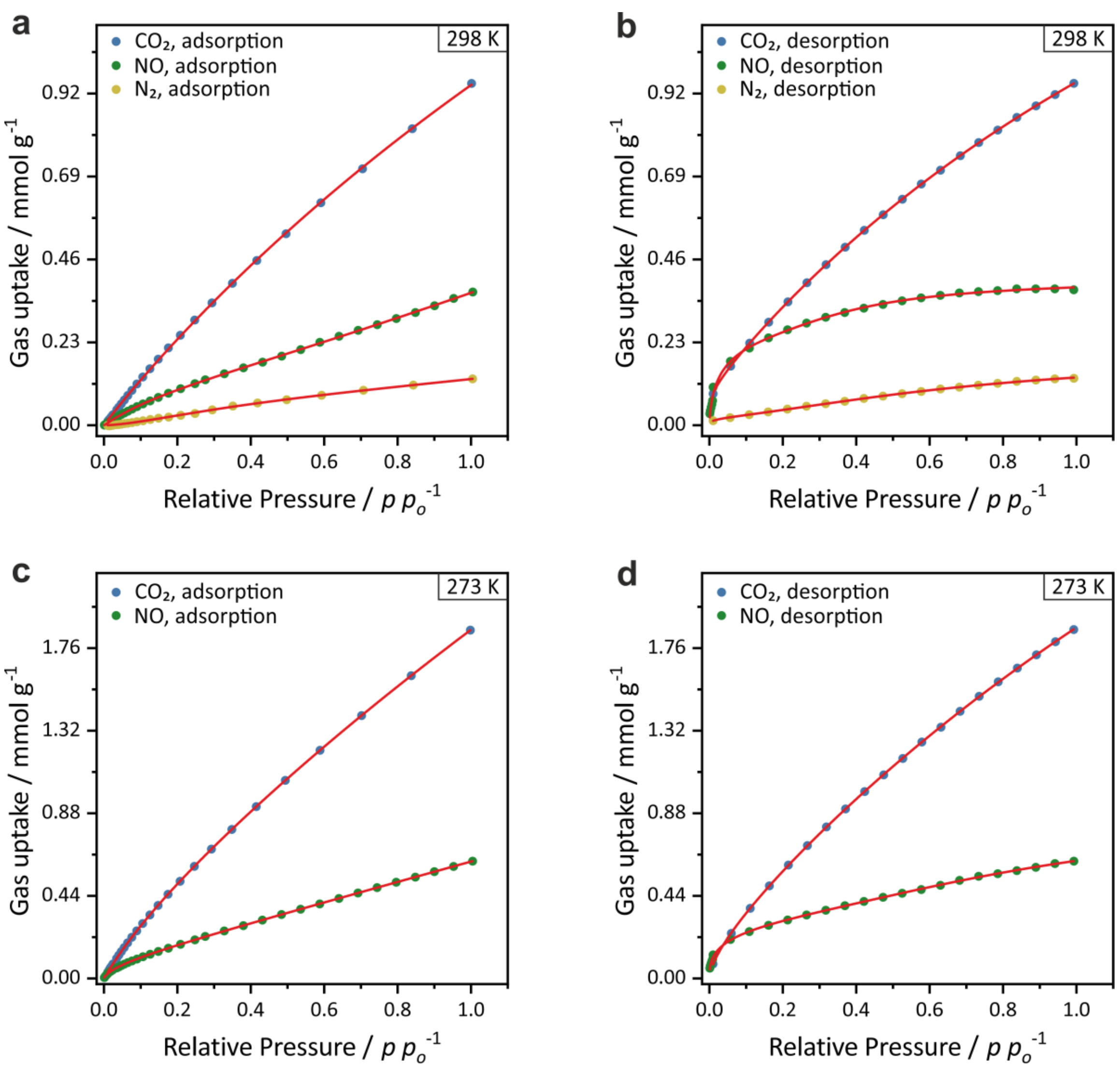

Figure S39. Fitting curves of **a** the adsorption isotherms at 298 K, **b** the desorption isotherms at 298 K, **c** the adsorption isotherms at 273 K and **d** the desorption isotherms at 273 K of TT-Imide-COF.

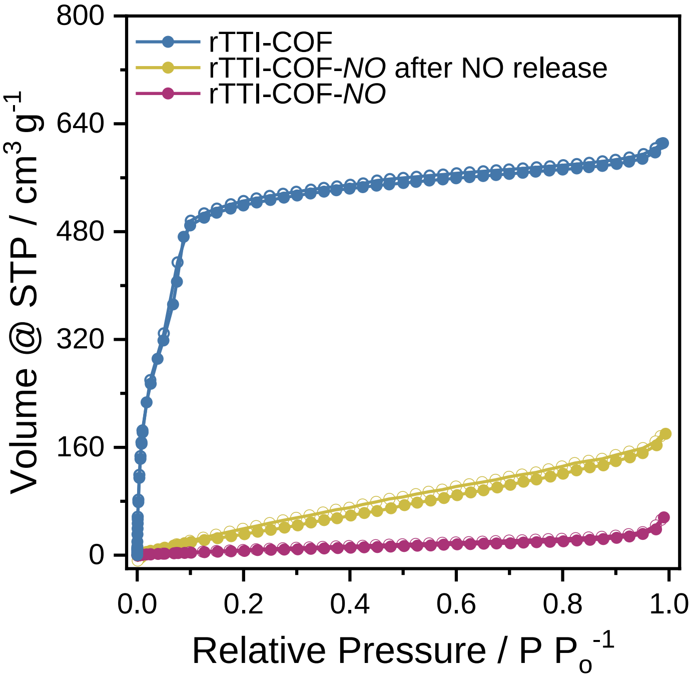

Figure S40. Argon adsorption isotherms at 87 K of rTTI-COF-*NO* (red) and rTTI-COF-*NO* after NO release (blue).

Supplementary XPS Spectra

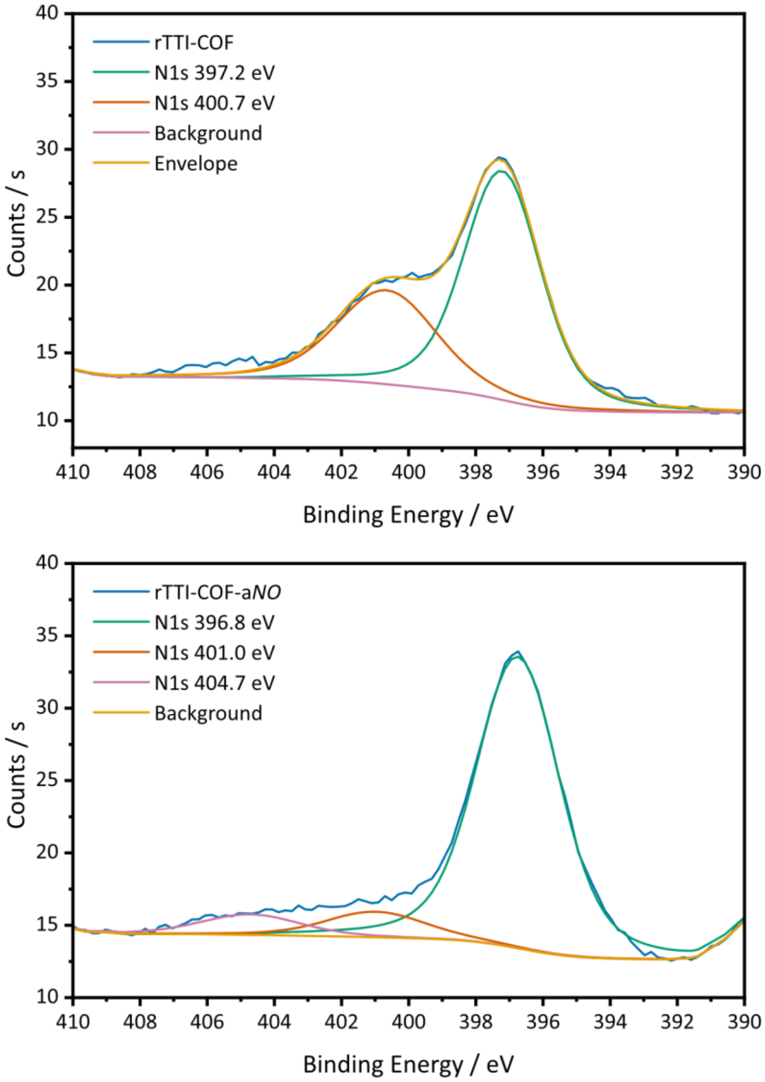

Figure S41. High-resolution N1s XPS spectra of rTTI-COF (top) and rTTI-COF-*aNO* (bottom). The N1s spectrum of the rTTI-COF was fitted with two peaks at 397.2 eV (triazine) and 400.7 eV (amine). The N1s spectrum of the rTTI-COF shows a significant decrease of the amine-shoulder correlating to the transformation of the secondary amine. It was fitted with three peaks at 396.8 eV (triazine), 401.0 and 404.7 eV (NONOate).[8,9] The low intensity of the NONOate signals is suspected to be the result of low stability of NONOates under strong X-rays.

Author Contributions

S. E. and J. M. initiated the project and carried out all experiments, analysis and data interpretation. B. V. Lotsch supervised the project. The manuscript was discussed and written through contributions of all authors.

⊥ S.E. and J.M. contributed equally to this work.

References

[1] J. W. M. Osterrieth, D. Fairen-Jimenez *et al. ChemRxiv. Prepr.* **2021**, https://doi.org/10.26434/chemrxiv.14291644.v1.

[2] J. Dong, Y. Wang, G. Liu, Y. Cheng, D. Zhao, *CrystEngComm* **2017**, 1*9,* 4899–4904.

[3] F. Haase, K. Gottschling, L. Stegbauer, L. S. Germann, R. Gutzler, V. Duppel, V. S. Vyas, K. Kern, R. E. Dinnebier, B. V. Lotsch, *Mater. Chem. Front.* **2017**, *1*, 1354–1361.

[4] L. Grunenberg, G. Savasci, M. W. Terban, V. Duppel, I. Moudrakovski, M. Etter, R. E. Dinnebier, C. Ochsenfeld, B. V. Lotsch, *J. Am. Chem. Soc.* **2021**, *143*, 3430–3438.

[5] F. Haase, E. Troschke, G. Savasci, T. Banerjee, V. Duppel, S. Dörfler, M. M. J. Grundei, A. M. Burow, C. Ochsenfeld, S. Kaskel, B. V. Lotsch, *Nat. Commun.* **2018**, *9*, 1–10.

[6] X. Zhu, S. An, Y. Liu, J. Hu, H. Liu, C. Tian, S. Dai, X. Yang, H. Wang, C. W. Abney, S. Dai, *AIChE J.* **2017**, *63*, 3470–3478.

[7] J. A. Hrabie, A. Srinivasan, C. George, L. K. Keefer, *Tetrahedron Lett.* **1998**, *39*, 5933–5936.

[8] A. Mohtasebi, T. Chowdhury, L. H. H. Hsu, M. C. Biesinger, P. Kruse, *Phys. Chem. C* **2016**, *120*, 29248−29263.

[9] Salmon, D. J., **2011**. Nitric Oxide- and Nitroxyl-Releasing Diazeniumdiolates in Pharmaceutical and Biomedical Reasearch Applications [Electronic PhD dissertation]. The University of Arizona. http://hdl.handle.net/10150/145389
